# Multiple sclerosis–associated CD226 variant disrupts vitamin D–induced type I Treg differentiation by impairing LFA-1 activation

**DOI:** 10.1172/JCI202193

**Published:** 2026-09-01

**Authors:** Saniya Kari, Aymeline Debonlier, Thibault Angles, Beatriz Chaves, Charles Grosjean, Florence Bucciarelli, Valérie Duplan-Eche, Lucie Nozeran, Jessica Lavery, Elena Morandi, Beatrice Pignolet, Max Mimpen, Joost Smolders, Roland Liblau, Abdelhadi Saoudi, Jan Damoiseaux, Frederick Masson, Loïc Dupré, Anne L. Astier

**Affiliations:** 1 Univ Toulouse, Toulouse Institute for Infectious and Inflammatory Diseases (Infinity), INSERM UMR1291 – CNRS UMR5051, Toulouse, France.; 2 School for Mental Health and Neuroscience, Maastricht University, Maastricht, Netherlands.; 3 MS Center ErasMS, Departments of Neurology and Immunology, Erasmus University Medical Center, Rotterdam, Netherlands.; 4 Central Diagnostic Laboratory, Maastricht University Medical Center, Maastricht, Netherlands.; 5 Medical University of Vienna, Department of Dermatology, Vienna, Austria.

**Keywords:** Autoimmunity, Immunology, Inflammation, Multiple sclerosis, T cells, Tregs

## Abstract

Multiple sclerosis (MS) is a complex inflammatory disease of the CNS resulting from an intricate interplay between genetic predisposition and environmental factors. Vitamin D (VD) deficiency is one of the established risk factors for MS. CD46 costimulation of CD4^+^ T cells induces a switch from Th1 to type I regulatory cells (Tr1), characterized by increased IL-10 production. This switch is impaired in MS T cells but can be restored by VD, which also strongly promotes expression of CD226 on CD46-activated T cells. The rs763361 polymorphism in the *CD226* gene, resulting in a nonsynonymous Gly307Ser variant, is associated with increased risk for MS. Herein, we show that expression of this *CD226* risk allele disrupts the ability of CD46-activated T cells to operate the IFN-γ/IL-10 switch upon VD exposure. Mechanistically, the risk variant impairs activation of the integrin LFA-1, which promotes the Tr1 phenotype. LFA-1–mediated Tr1 differentiation is also impaired in MS T cells expressing the *CD226* risk allele upon CD46 and VD stimulation. Our study unveils how, in the context of MS susceptibility, a genetic polymorphism and an environmental factor act in concert to control the differentiation of Tr1 cells.

## Abstract 2

Expression of a genetic risk variant associated to increased risk of multiple sclerosis alters the response of a T cell regulatory population to vitamin D.

## Graphical abstract

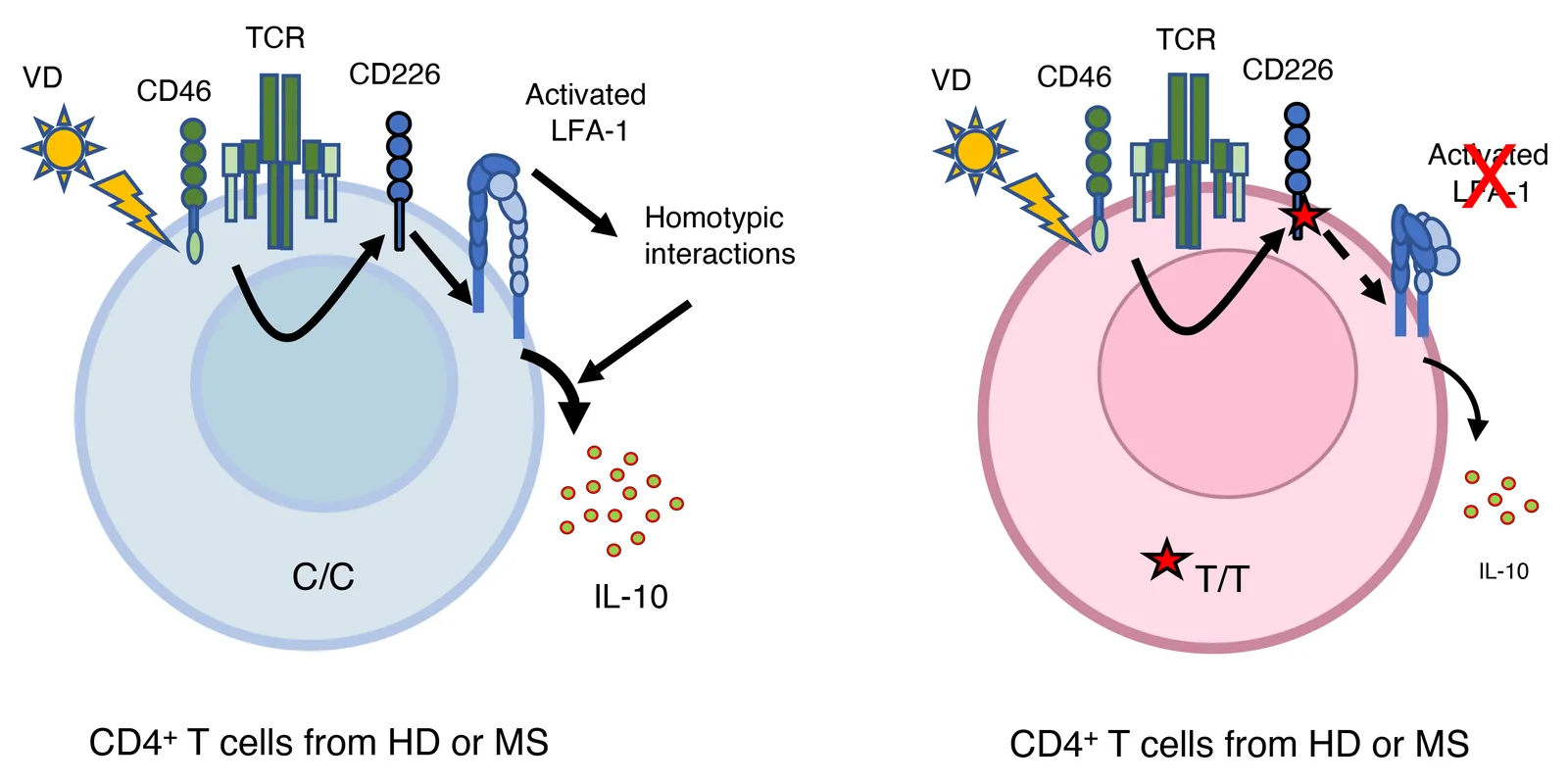

## Introduction

Multiple sclerosis (MS) is a chronic inflammatory disease of the CNS driven by a complex interplay between genetic predisposition and environmental triggers (1, 2). The exact cause of MS remains poorly understood. While several risk variants in immune genes have been identified, the mechanisms by which they interact with environmental factors to contribute to disease onset and progression in individual patients are still unclear. Among environmental factors, Vitamin D (VD) deficiency is a global health concern, and it is one of the most established modifiable risk factors for various inflammatory diseases, including MS (3). Persons with MS (pwMS) often exhibit VD deficiency and low VD levels have been also associated with increased disease severity and higher risk of disability (4).

CD4^+^ T cells play a pivotal role in MS, contributing through both effector T cells that recognize autoantigens and a dysfunction in Treg cells (5–8). In humans, the complement regulator CD46 acts as a costimulatory molecule on CD4^+^ T cells (9), influencing their differentiation. While first promoting Th1 differentiation, as IL-2 accumulates, CD46 switches human T cells from a Th1 to a type I regulatory T cell (Tr1) phenotype (10, 11). Tr1 are characterized by moderate IFN-γ and high IL-10 secretion and they play a crucial role in regulating immune responses and maintaining peripheral tolerance. The CD46-mediated Tr1 cell differentiation pathway is dysregulated in MS and other chronic inflammatory diseases. In these patients, CD46-costimulated T cells produce less IL-10, while maintaining normal levels of IFN-γ (6, 11–15). Mechanistically, CD46 ligation induces its proteolytic cleavage and CD46 recruitment to the immune synapse (IS) is required for the regulatory Tr1 switch (16).

VD plays an important role in the process of CD46-induced Tr1 differentiation. Exposure of CD4^+^ T cells to active VD (1,25OH, calcitriol) promotes the shift towards IL-10 production and partially restores the impaired CD46-mediated Tr1 switch in MS T cells, as evidenced by increased bystander suppression (17). This suggests that VD modulates CD4^+^ T cell differentiation, at least in part, by modulating the CD46 pathway (17–19). Indeed, we demonstrated the existence of a crosstalk between the VD and CD46 pathways by showing that CD46 costimulation led to increased expression of the VD receptor (VDR) and of the enzymes CYP27B1 and CYP24A1, key components of VD metabolism, in Tr1 cells (18). This CD46/VD crosstalk leading to increased VDR expression and switch from production of IFN-γ to IL-10 was further confirmed in patients with severe COVID-19 (19). Moreover, the phenotype of ex vivo T cells from pwMS supplemented with VD resembles that of CD46-costimulated Tr1 cells in vitro, suggesting the activation of the CD46 pathway in vivo (18). Among the phenotypic changes observed after exposure to VD either in vitro or in vivo, a strong increase in CD226 expression on CD46-induced Tr1 cells was noted, although less pronounced in MS, suggesting a potential role for CD226 in their function (18).

The costimulatory molecule CD226 plays a critical role in the differentiation of Th1 and Th17 (20–22). CD226 also serves as a marker of Tr1 cells when coexpressed with LAG3 and CD49b (23). The interaction between CD226 on Tr1 cells and its ligands on myeloid cells (CD155, CD112) is involved in the killing of antigen-presenting cells by the perforin/granzyme B pathway (24). This interaction also promotes survival, as Tr1 cells from mice deficient in CD226 are more prone to apoptosis and display reduced suppressive capacity (25). Upon T cell activation, CD226 associates with the integrin LFA-1 (CD11a/CD18), which enhances the costimulatory function of the latter (26, 27). Furthermore, the expression of CD226 is associated with LFA-1 conformational maturation (28), which is key to mediate firm T cell adhesion (29). Therefore, CD226 is a key molecular partner of LFA-1, sustaining its costimulatory properties, but also presumably its proadhesive function.

GWAS have reinforced the crucial role of the immune system in MS by identifying over 200 regions associated with increased MS risk, with most of the candidate genes involved in immune functions (30). Two SNPs in the gene encoding CD226 (rs727088 and rs763361) were identified as increasing risk of developing MS, as well as other chronic inflammatory diseases (31). The rs763361 variant (C>T), homozygous in approximately 25% of the population, leads to a Gly307Ser change in the intracellular domain of CD226. This variant has been shown to alter Foxp3^+^Treg cell suppressive activity (32), to promote IL-17 secretion by CD4^+^ T cells (21), to enhance IFN-γ secretion by CD8^+^ T cells (33) and to promote effector response in experimental autoimmune encephalomyelitis, a murine model of MS (34).

Given the impact of VD and CD226 on Tr1 differentiation and function, we explored the possibility that these 2 MS risk factors may tune Tr1 activity in a combined fashion. Deletion of CD226 by CRISPR/Cas9 in primary human CD4^+^ T cells reduced secretion of IL-10 production by CD46-costimulated T cells, demonstrating a role for CD226 in the activation of CD4^+^ T cells via CD46. Furthermore, T cells from healthy donors carrying the *CD226* risk allele displayed a reduced IFN-γ/IL-10 switch upon combined CD46 costimulation and VD exposure. Mechanistically, VD strongly induced LFA-1 conformational activation in CD46-coactivated T cells, but this effect was impaired in T cells expressing the *CD226* risk allele. VD-induced LFA-1 conformational activation was required for Tr1 differentiation and IL-10 production. Importantly, T cells from pwMS expressing the *CD226* risk variant also failed to activate LFA-1 upon VD exposure, which was associated with reduced Tr1 cell generation.

Taken together, these findings indicate that CD226 is pivotal for the generation of Tr1 cells in the context of CD46 costimulation and VD exposure. T cells expressing the *CD226* risk variant are unable to acquire the VD-induced Tr1 phenotype, which is explained by the inability to activate LFA-1. Our study therefore highlights how genetic and environmental factors may exert a combined control over the tuning of T cell function in the context of MS and potentially other pathologies with immune dysregulation.

## Results

### CD226 deletion reduces IL-10 production by CD46-costimulated CD4^+^ T cells.

Expression of CD226 is strongly upregulated by CD46-costimulation (18). To investigate the potential role of CD226 in the CD46 pathway, we knocked-out CD226 in primary human CD4^+^ T cells by CRISPR/Cas9. The cells were then activated by anti-CD3 or anti-CD3/CD46 antibodies and CD226 expression was assessed by flow cytometry. As expected, CD226 expression was strongly induced by CD46 costimulation in T cells transfected with the control guide (sgCtrl) (Figure 1, A and B). The deletion of CD226 with guide 1 (sgCD226-1) resulted in a strong but partial reduction in expression, while guide 2 (sgCD226-2) achieved a complete knockout (Figure 1, A and B). CD226 deletion did not affect the shedding of CD46 nor the induction of CD69 upon CD46-mediated activation (Figure 1, C and D). Additionally, CD226 deletion did not impact the percentage of Tr1 cells induced upon CD46 stimulation, as assessed by the co-expression of the activation markers CD49b and LAG3 (23) (Figure 1, E and F). Given that CD46 plays a key role in regulating the production of IFN-γ and IL-10 (10, 11), we next analyzed cytokine production in CD46-activated CD4^+^ T cells by flow cytometry by comparing T cells transfected with the control guide (sgCtrl) or the most effective guide targeting CD226, sgCD226-2 (Figure 1G). In the absence of CD226, an approximately 40% reduction average in the production of IL-10 was observed (Figure 1, G and H). Of note, a reduction in IL-10 production was observed using sgCD226-1, targeting a different *CD226* sequence (Supplemental Figure 1; supplemental material available online with this article; https://doi.org/10.1172/JCI202193DS1). We also assessed cytokine secretion in the supernatants by Cytometric Bead Array (CBA). *CD226* gene editing reduced cytokine production compared with sgCtrl-transfected T cells, with a more pronounced effect observed in IL-10 production, as shown by calculating the percentage of control values in cytokine production in response to VD (Figure 1I). Hence, these data are coherent with the data obtained by flow cytometry, showing a reduced production of IL-10 in T cells depleted in CD226. These data indicate that CD226 participates in the CD46 pathway by modulating cytokine production by activated cells.

We have previously reported that CD46 recruitment to the IS is necessary for its regulatory functions (16). Since CD226 is also recruited to the IS upon T cell activation (35) and associates with LFA-1 (27), we investigated whether CD226 contributes to CD46 recruitment using CRISPR/Cas9-mediated gene editing. Primary CD4^+^ T cells were transfected with the sgCtrl or sgCD226-2 guide and activated for 2 days. We next formed conjugates between the T cells and model antigen-presenting cells (36), consisting of P815 cells coated with anti-CD3/anti-CD46 Abs (Supplemental Figure 2, A–D). We used the ImageStreamX Mark II flow imager to quantify the expression and recruitment of CD46 and LFA-1 at the IS. As expected, CD226 expression was strongly reduced following sgCD226-2 transfection (Supplemental Figure 2E). CD226 deletion significantly reduced the recruitment of CD46 at the IS, while it had no significant impact on the recruitment of CD11a (Supplemental Figure 2F). Overall, these data further support the role of CD226 in controlling the CD46 pathway in primary CD4^+^ T cells.

### The CD226 rs763361 risk variant has no major impact on the CD46 pathway.

Since the *CD226* rs763361 variant (T/T) has been associated with an increased risk of developing MS (31), we next assessed whether this variant had an impact on the CD46-induced T cell activation pathway. To do this, we compared T cell responses induced by CD3/CD46 costimulation from a cohort of healthy donors that were homozygous for the nonrisk (C/C) or risk (T/T) genotype of *CD226*. T cell activation was not influenced by the *CD226* genotype, as the expression levels of activation markers, such as CD69, CD25, PD-1, and CD46, were similar between the 2 groups (Figure 2A). Likewise, the frequency of Tr1 cells did not show any marked difference between the 2 groups (Figure 2B). While no major differences in cytokine production were observed, there was a trend (*P* = 0.0525) toward an increased proportion of IFN-γ–producing cells in donors expressing the *CD226* risk variant (Figure 2C), which corresponded with the IFN-γ^+^IL-10^–^–producing cells (Figure 2D). These findings suggest that the *CD226* risk variant has only a modest impact on the CD46-induced T cell activation pathway.

### Modulation of IFN-γ/IL-10 production by 1,25OH is ineffective in T cells carrying the CD226 rs763361 risk variant.

Active VD (1,25OH) has been shown to reduce production of IFN-γ while maintaining IL-10 in CD46-costimulated T cells, thereby enhancing their suppressive function (17). Among the phenotypic changes observed, 1,25OH strongly increases CD226 expression on CD46-costimulated T cells, with a less pronounced induction in T cells from pwMS (18). To explore the impact of the *CD226* variant on T cell responses to VD, we assessed CD4^+^ T cells from healthy donors homozygous for either the C/C (nonrisk) or T/T (risk) genotype. These cells were costimulated with anti-CD3/CD46 antibodies in the presence or absence of 1,25OH. The phenotypic analyses revealed no major differences between the 2 groups (Supplemental Figure 3A). Although 1,25OH slightly increased the percentage of Tr1 cells in donors carrying the C/C genotype, this response was heterogeneous among donors. Analysis of the fold change induced by VD revealed no significant difference between the C/C and T/T genotypes in the modulation of Tr1 cell frequency. However, a lower proliferation rate was observed in risk allele–expressing cells when exposed to 1,25OH (Supplemental Figure 3A). Cytokine production was assessed at the cellular level by flow cytometry (Figure 3, A and B, and Supplemental Figure 3B), and in the supernatants (Supplemental Figure 3C). When cytokines were examined by flow cytometry, 1,25OH reduced IFN-γ production (Figure 3A and Supplemental Figure 3B) in T cells carrying the C/C or T/T genotypes. However, it also reduced IL-10 secretion in the T/T donors (Supplemental Figure 3B), which was consistent with the increased IL-10/IFN-γ ratio detected in the supernatants following 1,25OH treatment in donors expressing the C/C genotype but not those expressing the T/T genotype (Supplemental Figure 3C). We next determined the frequency of the T cells in the IFN-γ/IL-10 quadrants and calculated the fold change upon addition of VD (Figure 3B). Again, we observed a reduction of the frequency of T cells secreting IFN-γ in both the IFN-γ^+^IL-10^–^ and the IFN-γ^+^IL-10^+^ cells, for both genotypes. In contrast, addition of 1,25OH enhanced the frequency of IFN-γ^–^IL-10^+^ cells only in donors carrying the C/C genotype. This was correlated with a reduced expression of c-MAF, a transcription factor associated with IL-10 production, in T/T donors, while T-bet expression remained unaffected (Figure 3C). We also examined expression of Blimp-1, another transcription factor implicated in the regulation of IL-10 (37). A reduced expression of Blimp-1 was observed in donors carrying the T/T allele compared with C/C donors. Furthermore, the frequency of Blimp-1^+^ cells was increased upon addition of VD but only in donors carrying the nonrisk allele (Figure 3C). We also observed a correlation between IL-10 and expression of c-MAF and Blimp-1, emphasizing the role of these 2 transcription factors in IL-10 production by CD46-activated T cells (Figure 3D). The differences in VD response were not due to aberrant VDR expression in risk allele–expressing cells (Figure 3E). We also analyzed VDR nuclear translocation by determining its cytoplasmic and nuclear localization by confocal microscopy after 2 days of activation. As expected, VD promoted VDR translocation to the nucleus, with no significant differences between the 2 *CD226* genotypes (Supplemental Figure 4, A and B). To evaluate VDR transcriptional activity, we measured the expression of CYP24A1, a canonical VDR target gene. CYP24A1 expression was strongly induced by VD, with no significant differences between the 2 genotypes (Supplemental Figure 4C). Together, these findings indicate that neither VDR nuclear translocation nor its transcriptional activity is altered in T cells carrying the *CD226* risk variant. We also deleted *CD226* by CRISPR/Cas9 in T cells homozygous for either the C/C or T/T variant and compared response with VD (Supplemental Figure 5). Deletion of *CD226* was similarly effective in both groups of donors (Supplemental Figure 5A). We determined production of cytokines when activated in the presence of VD (Supplemental Figure 5, B and C), and, to normalize the data, we calculated the percentage of control values (deletion of *CD226* compared to control guide) (Supplemental Figure 5D). As expected, deletion of the C/C variant systematically (except for one donor) reduced IL-10 production, as well as the IL-10/IFN-γ ratio, while there was no significant impact on T/T donors. Production of IFN-γ and IL-17 was not affected in T cells carrying either genotype. Hence, only targeting of *CD226* in the C/C donors reduced IL-10 production, further emphasizing the importance of the C/C allele in driving IL-10 production, and the altered function of the T/T variant. We next determined whether the effect observed with the *CD226* T/T variant on IL-10 production was also observed within the Tr1 population by assessing cytokine production within the CD49b^+^LAG3^+^ gated population, the population secreting the most IL-10 (Supplemental Figure 6). VD significantly increased IL-10 production in Tr1 cells from donors carrying the C/C allele, whereas this was not observed in donors carrying the T/T variant. In contrast, VD-mediated reduction in IFN-γ was observed for both genotypes. Overall, these data show that the effect of CD46 costimulation combined with 1,25OH exposure to favor IL-10 production over that of IFN-γ is impaired in Tr1 cells expressing the *CD226* risk variant.

### The CD226 rs763361 risk variant does not impact early signaling in the presence of VD.

Although most effects of VD are mediated through VDR-dependent regulation of gene expression, VD can also exert rapid nongenomic effects, including the modulation of intracellular signaling pathways and protein phosphorylation (38). Having identified an altered cytokine response to VD in T cells carrying the *CD226* risk allele, we therefore investigated whether early signaling events triggered by CD46 costimulation differed between the 2 *CD226* genotypes. We first assessed recruitment of CD46, CD226, and LFA-1 at the IS on T cells from donors expressing either the *CD226* nonrisk or risk allele by forming conjugates between primary CD4^+^ T cells and P815 cells coated with anti-CD3/anti-CD46 Abs (Figure 4A). We quantified the recruitment of CD46, CD226, and LFA-1 at the IS in the absence or presence of VD on a large number of conjugates in 4 donors carrying the C/C or T/T allele (ranging from 76–100 conjugates analyzed per donor per condition). No significant effect of the *CD226* genotype was observed, although a trend towards a reduced recruitment of CD46 was observed in donors expressing the *CD226* risk allele (Figure 4B). Preincubation with 1,25OH did not substantially impact the IS recruitment patterns of CD46 and LFA-1 between the 2 genotypes (Figure 4B). Overall, these data show that neither the risk allele nor 1,25OH significantly impact the recruitment of CD46 and LFA-1 at the IS. We next analyzed whether early CD3/CD46 signaling was affected in CD4^+^ T cells expressing either the C/C or T/T *CD226* genotype by assessing phosphorylation of Tyrosine, ZAP70, and ERK (Figure 4C). 1,25OH reduced levels of tyrosine phosphorylation in both groups, had no marked impact on ZAP70, and reduced phosphorylation of ERK in C/C-expressing T cells. However, no difference in the fold change of the phosphorylation of these 3 proteins between the 2 variants was observed upon addition of 1,25OH. Hence, the risk allele does not significantly modulate the early signaling events in CD3/CD46-activated T cells in response to 1,25OH. Therefore, this does not appear to account for the differential response to VD observed in downstream functional readouts.

### LFA-1 conformation activation by CD46/1,25OH supports Tr1 conversion and is impacted by the CD226 rs763361 risk variant.

Given the physical association of CD226 to LFA-1 (26), we asked whether the ability of CD226 to favor Tr1 cell development might be related to LFA-1 activation and function. We first investigated whether the T/T risk variant impacted LFA-1 conformational maturation, using the high affinity–specific m24 antibody (39). As compared with CD28 costimulation, CD46 costimulation strongly promoted LFA-1 activation (Figure 5, A and B). However, activation of LFA-1 was significantly reduced in T cells expressing the *CD226* risk variant. Similarly, in CD4^+^ T cells deleted in CD226 by CRISPR/Cas9, CD46-induced LFA-1 activation was severely reduced (Supplemental Figure 7). Thus, the *CD226* T/T risk variant does not appear to sustain the conformation activation of LFA-1 in the context of T cell activation via the TCR and CD46. We next tested the impact of the T/T variant on LFA-1 activation in the context of the combined CD46/VD stimulation. Remarkably, addition of 1,25OH to T cells expressing the C/C genotype led to a major increase in LFA-1 conformational activation (Figure 5, C and D). This was not observed in T cells expressing the T/T genotype, despite similar expression levels of CD11a across both genotypes (Figure 5, C and D). Hence, VD stimulates potent LFA-1 activation upon costimulation via the TCR and CD46, but this activity is impaired in cells carrying the *CD226* risk allele. We also examined LFA-1 activation in the gated Tr1 population (Supplemental Figure 8). Activation of LFA-1 was induced by addition of VD in Tr1 cells from both genotypes although the effect reached significance only in C/C donors. The high level of LFA-1 activation observed in Tr1 cells is likely related to their activated phenotype, as indicated by their high expression of CD25 (Supplemental Figure 8). Next, to test whether LFA-1 activation may contribute to the process of Tr1 differentiation, we stimulated T cells from C/C donors with anti-CD3/CD46 antibodies and 1,25OH in the presence of the conformation-specific LFA-1 antagonist BIRT377 (40). Blocking LFA-1 activation did not affect cell viability, though a slight reduction was observed with BIRT377 and VD (Supplemental Figure 9A). As expected, LFA-1 activation was inhibited upon BIRT377 treatment (Supplemental Figure 9A). Blocking LFA-1 activation led to a reduction in the development of Tr1 cells (Figure 5E). Addition of BIRT377 significantly reduced the secretion of IL-10 in the presence of 1,25OH without affecting IFN-γ levels (Supplemental Figure 9B). Furthermore, while VD increased the proportion of IFN-γ^–^IL-10^+^ T cells, this was not observed in the presence of BIRT377 (Figure 5F). A reduced proportion of IFN-γ^+^IL-10^+^ T cells with VD in the presence of BIRT377 was also observed, as also noted when comparing the fold change in the presence of VD. These data indicate that LFA-1 inhibition impairs the VD-induced increase in IL-10–producing T cells. This was accompanied by reduced expression of T-bet (Figure 5G). We next examined production of IL-10 and IFN-γ by the gated Tr1 population (Supplemental Figure 9C). As observed with T cells, addition of BIRT377 reduced the ability of VD to increase the proportion of IFN-γ^–^IL-10^+^ Tr1 cells. These data indicate that LFA-1 conformational activation is mediating a process that sustains Tr1 differentiation upon stimulation of T cells via the TCR and CD46 in the presence of 1,25OH.

### LFA-1 dependent homotypic adhesion sustains IL-10 production in the context of CD46/VD stimulation.

We next explored the nature of the process by which LFA-1 may sustain Tr1 differentiation. As LFA-1 is key to mediate homotypic T cell interactions (41, 42), we examined whether these interactions might be required to sustain IL-10 production. CD46-activated T cells from C/C donors were stimulated with anti-CD3/CD46 antibodies and 1,25OH, and their cell clusters were disrupted twice daily. Mechanical disruption of these cell clusters did not impact LFA-1 activation (Figure 6A). Although no significant differences in IFN-γ or IL-10 production were detected, likely due to interdonor variability (Figure 6B), cluster disruption consistently reduced the IL-10/IFN-γ ratio in the presence of VD (Figure 6C). These data suggest that T cell homotypic interaction mediated by LFA-1, rather than LFA-1 activation itself, is conditioning the switch toward a Tr1 phenotype. To further examine the LFA-1–driven adhesive properties of CD46/VD stimulated T cells, these cells were imaged upon interaction with ICAM-1. T cells from donors carrying either the C/C or T/T *CD226* genotype were prestimulated via the TCR and CD46 in the presence or absence of 1,25OH. These cells were then incubated for 20 minutes on precoated ICAM-1 wells, stained for F-actin and p-Tyr, and imaged with an automated confocal device (Figure 6, D and E). To directly compare the response to VD between genotypes, the percentage change induced by 1,25OH relative to untreated controls was calculated for each donor and compared between the C/C and T/T groups. T cells carrying the C/C but not those with the T/T genotype appeared to remodel their actin cytoskeleton upon 1,25OH exposure, as measured by the redistribution of F-actin toward the center of the contact area (actin profile 5) (Figure 6E and Supplemental Figure 10). Although these changes are modest, they suggest that the *CD226* risk variant alters actin remodeling following LFA-1 engagement with ICAM-1, a process relevant to T cell homotypic interactions. While tyrosine phosphorylation was maintained in the presence of VD in T cells from donors carrying the nonrisk allele and reduced in T cells from donors expressing the T/T genotype, the VD-induced fold change did not significantly differ between the 2 genotypes (Figure 6E).

### Expression of the CD226 risk variant alters LFA-1 activation in T cells from pwMS.

Having previously described that the CD46 pathway is dysregulated in MS (6), we next assessed the impact of the *CD226* risk allele in CD46-costimulated T cells from pwMS. CD4^+^ T cells from patients with RRMS carrying either the C/C or T/T genotype were activated by anti-CD3/CD46 antibodies in the presence or absence of 1,25OH and the phenotype of the cells was examined by flow cytometry. While HD carrying the *CD226* risk allele (T/T) showed minimal differences in T cell activation and Tr1 differentiation compared with nonrisk allele carriers (C/C) (Figure 2), patients with RRMS with the T/T risk genotype demonstrated notable impairments in T cell activation when stimulated with anti-CD3/CD46 antibodies. Specifically, CD4^+^ T cells from pwMS expressing the risk allele exhibited lower levels of activation and fewer Tr1 cells than C/C carriers (Figure 7, A and B). VD had no major impact in the overall phenotype of these CD46-costimulated T cells from pwMS (Figure 7B). However, we found a difference in LFA-1 activation. Similar to the findings in HD, 1,25OH significantly boosted LFA-1 activation in C/C genotype carriers, but this effect was reduced in T cells from T/T carriers (Figure 7C). We next assessed cytokine production in both CD4 and Tr1 cells from pwMS (Figure 7, D and E). Because some T/T MS carriers displayed lower activation levels than C/C carriers (Figure 7B), we also analyzed cytokine production within the gated Ki67^+^ cell population (Figure 7F). VD significantly reduced the proportion of IFN-γ^+^IL-10^–^ and IFN-γ^+^IL-10^+^ CD4^+^ T cells in both genotypes, with a trend towards increased IFN-γ^–^IL-10^+^ T cells in C/C carriers. A significant reduction in the proportion of IFN-γ^+^IL-10^–^ was observed in Tr1 cells for both genotypes, with a significant increase in IFN-γ^–^IL-10^+^ in the C/C donors only. Comparable findings were obtained in the Ki67^+^ population. Although the cohort size remains limited, these data suggest that T/T MS carriers may also produce less IL-10 than C/C carriers in the presence of VD.

This impairment in LFA-1 activation, seen in both HD and pwMS with the T/T genotype, underscores how the risk allele modulates the VD response in relation to LFA-1 activation, implying that the therapeutic effects of VD supplementation may partially depend on this genotype. As a proof of concept, pwMS treated by IFN-β and supplemented by VD or placebo for 48 weeks from the SOLARIUM trial (43) were stratified according to the *CD226* risk allele when genetic material was available (44) (Supplemental Figure 11). MRI activities were independent of the genotype of the patients in the placebo group. In contrast, they were statistically differently distributed among the genotypes of the patients in the VD-supplemented group, as the 5 pwMS homozygous for the nonrisk allele and the 11 heterozygous individuals had no MRI activity, while the only 2 pwMS with MRI activities were homozygous for the *CD226* risk allele (Supplemental Figure 11). Although not conclusive, considering the limited number of patients that could be studied, these data support the hypothesis that the *CD226* allele may impact response to VD in pwMS.

## Discussion

While individual environmental and genetic factors have been associated with an increased risk for MS, how these multiple risk factors interact to regulate the immune response in MS is still ill-defined. Herein, we demonstrate that a MS risk variant in *CD226* hinders the activity of VD toward the differentiation of a regulatory T cell population known to be critical for the control of MS. This variant was already reported to alter Foxp3^+^ Treg cell suppressive function and to promote CD4^+^ and CD8^+^ effector T cell functions (21, 33, 34). We now demonstrate that this variant also alters the regulatory CD46 pathway in human CD4^+^ T cells, by dysregulating the balance between production of proinflammatory IFN-γ and regulatory IL-10 in the presence of 1,25OH. We could not detect any substantial amount of TGF-β secreted by these cells, consistent with previously reported data (10). VD strongly reduced IFN-γ production, consistent with previous studies demonstrating that VD modulates the IL-12/IFN-γ axis through inhibition of JAK/STAT signaling (4). Notably, this effect was observed irrespective of the *CD226* genotype, suggesting that the genotype-dependent effects described here primarily influence the acquisition of the IL-10–producing phenotype rather than the suppression of Th1 responses. Importantly, although some of the changes observed with the risk variant have modest effect size, such effects are consistent with the biology of MS genetics. Indeed, with the exception of HLA-DRB1*15:01, most MS-associated variants confer relatively small effect sizes (e.g., *CD226* odds ratio is 1.12) (31). These variants are thought to contribute to MS susceptibility in combination with others and in interaction with environmental factors, as shown herein in our study, by demonstrating a gene-environment interaction between the *CD226* variant and VD.

We had previously shown that recruitment of CD46 at the IS was necessary for the IFN-γ/IL-10 switch (16). Herein, addition of 1,25OH had no major impact of the recruitment of CD46 to the IS. Phosphorylation of Tyrosine and ERK upon CD46 costimulation was reduced upon addition of 1,25OH, in line with a previous report (45) but there was no difference between the 2 variants. Mechanistically, we show that expression of the risk allele impaired activation of LFA-1. While this was observed in the absence of VD, the impaired LFA-1 activation was more marked in the presence of 1,25OH. LFA-1 activation was previously shown to be reduced in a subset of CD8^+^ T cells lacking CD226 expression (28). Our data demonstrate the key role of CD226 in activation of LFA-1, as deletion of CD226 by CRISPR/Cas9 reduced LFA-1 conformational activation observed upon CD46 costimulation. High-affinity maturation of LFA-1 relies on engagement with ICAM-1 and inside-out signaling originating from the TCR or chemokine receptors (46, 47). Furthermore, LFA-1 ligation can also promote T cell activation via outside-in signaling (46, 48). The significant reduction in LFA-1 activation upon CD3/CD46 costimulation observed in T cells from healthy individuals expressing the risk allele is in line with the role of CD226 in sustaining the inside/out signaling leading to the activation of LFA-1 and its enhanced affinity for ICAM molecules. It suggests that the *CD226* risk variant might be dysfunctional as sustaining the inside/out signaling leading to the conformation activation of LFA-1. Our data show that the T/T variant does not significantly impact the IS recruitment of CD46, CD226, and CD11a upon addition of VD, nor the ignition of early phosphorylation events (p-Tyr, p-ZAP70, and p-ERK). However, it is possible that the inside-out signaling pathway leading to LFA-1 activation is impacted by the risk variant, and this will require further investigation. LFA-1 regulates T cell homotypic interactions, previously shown to control T cell differentiation by promoting a regulatory phenotype (42) and modulating effector functions of T cells (49). Homotypic interactions also allow sharing of cytokines between primed T cells (50), including IL-2 (41), which may contribute to the CD46-mediated IFN-γ/IL-10 switch. The existence of homotypic T cell interactions has also been documented in vivo (50), and clustering of Treg cells is key to maintain immune homeostasis (51). By using an allosteric antagonist of LFA-1, we show that LFA-1 activation is required to promote IL-10 production by CD46-activated T cells in response to VD. Activation of LFA-1 in the presence of VD allowed actin remodeling within the cells, which was impaired in T cells from donors expressing the *CD226* risk variant. These data suggest that, in the presence of VD, signaling events induced upon ICAM binding are altered with the T/T variant, resulting in aberrant actin localization, reducing in fine IL-10 production. Upon ICAM-1 binding, T cells carrying the C/C variant tend to display enriched F-actin toward the nucleus mask (Actin Profile 5/5, Supplemental Figure 10). It is mildly increased upon addition of VD in the T cells from the donors carrying the C/C genotype, while no changes are observed in donors carrying the T/T variant. This compartment might correspond with the central pool of actin recently reported (52) that maintains cellular coherence and protects the nucleus during migration in confined environments. We may speculate that remodeling of this compartment by VD in differentiating Tr1 cells is important for the assembly of homotypic aggregates and the associated cell:cell communication. Indeed, mechanical disruption of the clusters led to a reduced IL-10/IFN-γ ratio of the cytokines secreted in the supernatants, but did not impede LFA-1 activation, indicating that the physical interactions between T cells rather than LFA-1 activation itself was key in driving the Tr1 phenotype. Of note, the *CD226* risk variant also impacted cytokine production by the gated Tr1 (CD49b^+^LAG3^+^) population, as it impaired the VD-induced increase in IL-10 production. Furthermore, the reduced IL-10 production observed in the presence of the BIRT377 inhibitor indicates that LFA-1 activation also contributes to the regulation of cytokine production by Tr1 cells in response to VD.

Interestingly, increased expression of CD226 has been reported on Tr1 cells from patients with another neuroinflammatory disease, neuromyelitis optica spectrum disorder (NMOSD) (53), suggesting a role for CD226 in their function in these patients as well. We recently reported increased levels of sCD226 in the serum of patients with MS and NMOSD (54). Whether CD226 is more or less shed according to the variants remains to be determined. Nevertheless, this also indicates a dysregulated CD226 pathway in these patients and the potential impact of the *CD226* risk allele.

Although our study is focused on MS, we uncover mechanisms governing Tr1 cell function that may be applicable to other chronic inflammatory diseases, in addition to MS, as the *CD226* variant is associated with risk for rheumatoid arthritis, type 1 diabetes, systemic lupus erythematosus, among other (31), diseases also associated with VD deficiency. Considering the undisputed data on VD deficiency and MS incidence and severity, VD supplementation trials have been performed but the results are not consistent (55). However, the recent results of a large randomized double-blind placebo-controlled trial (D-Lay-MS) aiming at supplementing patients with high dose of VD as a monotherapy at their first clinical symptoms (trial NCT01817166) show that VD significantly reduced disease activity in early MS (56). We show that the in vitro Tr1 response to VD in donors expressing the *CD226* risk allele is altered, implying that the therapeutic effects of VD supplementation may partially depend on this genotype. By stratifying patients from the Solarium VD supplementation trial (NCT01285401) (43) according to their *CD226* genotype, we observed that only patients carrying the T/T genotype had MRI activity upon VD supplementation. Although only a limited number of patients could be genotyped and studied, these preliminary data support the hypothesis that the *CD226* allele may impact response to VD in pwMS, and that stratification of pwMS according to their *CD226* allele may clarify the role of VD supplementation in MS.

Overall, we uncover how the *CD226* rs763361 variant and VD combined control a key regulatory T cell pathway altered in MS, illustrating an interaction between a genetic polymorphism and an environmental factor both associated with increased risk for MS.

## Methods

### Sex as a biological variable.

As there is a strong sex bias in MS, with a ratio of 3 females for 1 male, all experiments using healthy donors have been done using female donors to avoid a confounding effect of the biological sex in the analysis of the effect of the *CD226* variant in Tr1 function.

### Healthy donors and pwMS.

PBMC were isolated from blood obtained after informed consent of donors from the immuno-monitoring platform at Infinity, Toulouse, France (Table 1). We genotyped the presence of the *CD226* SNP rs763361 using the TaqMan SNP Genotyping Assay (Applied Biosystems) according to manufacturer’s protocols. We only analyzed homozygous individuals expressing the rs763361T risk genotype (CD226307Ser, T/T), or the nonrisk rs763361C genotype (CD226307Gly, C/C). The two healthy donor subgroups (C/C versus T/T genotypes) had a comparable mean age: (C/C 39,56 and range 18–59 years old (y.o.); T/T 38,89, range 19–64 y.o.) (Table 1). PBMC from pwRRMS (mostly diagnosed according to the 2005 revisions of McDonald criteria) were selected from either the BIONAT cohort (ClinicalTrials.gov registration number: NCT00942214) or the NEUROBANK cohort (Table 2). For the BIONAT cohort, PBMC were selected before Nataluzimab treatment initiation and after a washout period from last treatment.

### Cell culture and stimulation.

Thawed PBMC were incubated for 3 hours or overnight at 37°C with 5% CO_2_ in complete culture medium RPMI 1640 with Glutamax (Gibco), or in Immunocult (StemCell) for Tr1 analysis, supplemented with sodium pyruvate (1mM, Gibco), penicillin (50 U/mL, Gibco), streptomycin (50 μg/mL, Gibco), Hepes (10 mM, Gibco), non-essential amino acids and 10% fetal bovine serum (FBS Sigma-Aldrich) before isolation of CD4^+^ T cells. After resting, PMBC were filtrated with 100 μM MACS SmartStrainers (Miltenyi Biotec). The negative selection of CD4^+^ T cells was performed using the Mojosort Human CD4 T cell isolation kit (Biolegend) or Easysep human CD4^+^ T cell isolation kit (Stemcell). The same purity (> 96%) and yield were obtained with both kits and there was no difference for the downstream application. We checked that the C/C and T/T donors had comparable distribution of naïve (CD45RA^+^) and memory (CD45RO^+^) cells (Supplemental Figure 12). Purified CD4^+^ T cells (1 × 10^6^ cells/mL) were activated with pre-coated anti-CD3 (clone OKT3, 5 μg/mL, Biolegend, Cat# 317326) and anti-CD46 (clone MC120.6 10 μg/mL, hybridoma kindly given by Mathias Faure) in the presence of rhIL-2 (10 U/mL, Peprotech). To homogenize the final IgG concentration used, irrelevant IgG1 (Biolegend) was added to the wells as required. At the beginning of the culture, cells were treated with 100 nM active VD (1,25OH) (calcitriol, Sigma) or the vehicle control, ethanol. In some experiments, 10 μM of the inhibitor of LFA-1 (Birt377, R&D Systems) was added.

### Inactivation of CD226 by CRISPR/Cas9.

Guides were selected using the Alt-R CRISPR-Cas9 guide RNA design checker tool (IDT, https://eu.idtdna.com/site/order/designtool/index/CRISPR_SEQUENCE) as well as CCTOP (https://cctop.cos.uni-heidelberg.de/) based on predicted on-target activity and minimal off-target potential. For each sgRNA, 1.5 nmol were dissolved with 15 μL of nuclease-free buffer (100 μM). The ribonucleoprotein (RNP) complex was prepared by incubating CD226 sgRNA (0,3 nmol, Synthego) with Cas9 nuclease (IDT). Pellets of isolated CD4^+^ T cells (from donors expressing the nonrisk C/C *CD226* genotype) (4 × 10^6^ cells/condition) were first mixed with 20 μL of nucleofector solution (P3 Primary Cell 4D Nucleofector X Kit S, Lonza) and then 5 μL of RNP complex: sgCtrl (5′-GCACUACCAGAGCUAACUCA-3′), sgCD226-1 (5′ GUUCAAGAUCGGGACCCAGC 3′) and sgCD226-2 (5′GUUAAGAGGUCGAUCUGACG 3′). The cell suspension was then transferred into a 16-well Nucleocuvette Strips and placed into an 4D-nucleofector X-unit device (Lonza). The program “T cell, human, unstimulated high efficiency” was selected. After nucleofection, the cells were cultured with complete culture medium for a minimum of 3 hours at 37°C before undergoing stimulation. CD4^+^ T cells were then stimulated as previously described with anti-CD3 and CD3/CD46 antibodies in presence of 1,25OH or the vehicle control. The knockout efficiency of CD226 and cell viability analysis was assessed by flow cytometry.

### Flow cytometry.

CD4^+^ T cells were stained with a live-dead marker (Zombie NIR, Biolegend, Cat# 423106) before surface staining was performed by incubating the cells with the following antibodies: CD49b-FITC (clone P1E6-C5, Biolegend, Cat# 359306), CD49b-APC (clone P1E6-C5, Biolegend, Cat# 359310), CD49b-BUV661 (clone AK-7, BD Bioscience, Cat# 750068), CD25-PerCP-Cy5.5 (clone BC96, Biolegend, Cat# 302626), PD-1-BV421 (clone EH12.2H7, Biolegend, Cat# 329920), CD11a-BV785 (clone HI111, BD Bioscience, Cat# 742626), CD226-PE-Dazzle 594 (clone 11A8, Biolegend, Cat# 338318), CD46-PE-Cy7 (clone Tra-2-10, Biolegend, Cat# 352408), CD46-FITC (clone MEM258, Biolegend, Cat# 315304) LAG3-PE-Cy5 (clone 11C3C65, Biolegend, Cat# 369346), LAG3-PE (clone 11C3C65, Biolegend, Cat# 369306), LAG3-BUV395 (clone T47-530, BD Bioscience, Cat# 569247), CD69-APC (clone FN50, Biolegend, Cat# 310910) in staining Buffer (2% FBS in PBS) for 30 minutes at 4°C in the dark. An example of staining for each marker is shown in Supplemental Figure 13. For intracellular staining, cells were fixed and permeabilized for 30 minutes at room temperature (Foxp3/transcription factor staining buffer set, eBioscience). Cells suspensions were incubated with the following antibodies: KI67-BV650 (clone B56, BD Bioscience, Cat# 563757), c-Maf-PerCPfluor710 (clone sym0F1, Thermo Fisher Scientific, Waltham, USA, Cat# 46-9855-42), T-bet-BV785 (clone 4B10, Biolegend, Cat# 644835), Blimp-1-APC (clone 646702, R&D, Cat# IC36081A), VDR-AF 647 (clone D-6, Santa Cruz biotechnology, Cat# sc-13133), IL-17A-BV421 (clone BL168, Biolegend, Cat# 512322), IFN-γ-BV605 (clone B27, Biolegend, Cat# 506542), IFN-γ-FITC (clone B27, Biolegend, Cat# 506504) and IL-10-PE (clone JES3-9D7, Biolegend, Cat# 501404) overnight at 4°C. Cells were analyzed using standardized panels with a FortessaX20 (BD Biosciences) or a Symphony A5 (BD Biosciences). After 2 days of stimulation, intracytoplasmic expression of cytokines was assessed after 4 hours stimulation with PMA (50 ng/mL, Sigma), ionomycin (500 ng/mL, Sigma) in the presence of monensin (2 μM, BD Bioscience) or brefeldin A (1 μg/mL, BD Bioscience). The cells were stained as described above. An example of staining for these markers is shown in Supplemental Figure 14. The flow cytometry results were analyzed using FlowJo v10.8 Software (BD Life Sciences).

### Quantification of cytokines by cytometric bead arrays.

Cytokines in the supernatants from d5 were quantified by a cytometric bead assay (LEGENDplex “*Human Th17 Panel”* (7-Plex), Biolegend) according to manufacturer’s recommendations. The quantification was performed using MACSQuant10 (Miltenyi Biotec). An acquisition of 300 events per analyte was done. The collected data were analyzed using Biolegend software (Cloud version 2024-06-15). The concentrations of cytokines were determined using a standard curve generated in the same assay.

### Recruitment at the immune synapse by imaging flow cytometer.

10 × 10^6^ P815 cells (murine mastocytoma cell line, ATCC: TIB-64) were pre-labeled with Cell Trace Violet (CTV, 0.3 μM, ThermoFisher Scientific) for 10 minutes at 37°C. 0.5 × 10^6^ cells were pre-coated with anti-CD3 (clone OKT3, 5 μg/mL, Biolegend) and anti-CD46 (clone MCI 20.6, 3.3 μg/mL) for 1 hour at 37°C to allow antibody binding to the Fc receptors. 0.5 × 10^5^ CTV^+^ coated P815 cells were then mixed with 1 × 10^5^ CD4^+^ T cells, which had been pre-incubated overnight with 100 nM 1,25OH (Sigma-Aldrich), in a 96-well U-bottom plate at a ratio of 2 CD4^+^ T cells for 1 P815 cell for 15 minutes at 37°C. These conjugates were labeled with the following antibodies: CD46-FITC (2 μg/mL, clone MEM258, Biolegend, Cat# 315304), CD11a-PE (2 μg/mL, clone HI111, Biolegend, Cat# 301208), and CD226-AF67 (1 μg/mL, clone DX11, BD Bioscience, Cat# 564797) for 10 minutes at 37°C. Cells were then fixed with 1% paraformaldehyde (PFA) for 10 minutes at 37°C. The conjugates were acquired at 40X magnification using the ImageStream Mark II Amnis flow cytometer (Amnis-EMD Millipore). The acquisition was performed at a low speed to achieve high image sensitivity. The data were analyzed using IDEAS 6.2.64.0 software (EMD Millipore). First, double-positive conjugates for CTV and CD11a (expressed only on CD4^+^ T cells) were selected at the focus (Supplemental Figure 2A). Only conjugates containing a single P815 cell and a single CD4^+^ T cell were chosen. Then, a mask was created to determine the IS area between the CD4^+^ T cell and the P815 cell (Supplemental Figure 2B). The median pixel intensity of CD46, CD226, and CD11a was calculated at the IS and on the CD4^+^ T cell. To normalize the data, we calculated the ratio of median pixel intensity at the interface by the median pixel intensity on the CD4^+^ T cell (Supplemental Figure 2C).

### Activated LFA-1.

After 5 days of stimulation, activation of LFA-1 was measured with anti-CD11a/CD18 antibody (clone m24, Biolegend, Cat# 363406) binding specifically the high affinity conformation of LFA-1. The cells were incubated with antibody in pre-warm complete culture medium with 2mM MgCl2 for 30 minutes at 37°C. The reaction was stopped with PBS containing 2% FBS and 2mM EDTA. The cells were analyzed the same day with a FortessaX20 (BD Biosciences). An example of staining is shown in Supplemental Figure 13E.

### Analysis of early TCR costimulation signaling.

Experiments were performed in 96-well round-bottom plates and by keeping the cells at 4°C. After overnight preincubation with 1,25OH or vehicle control, CD4^+^ T cells (2,5 **×** 10^5^ cells per well) were left unstimulated or stimulated with 50 μL of soluble anti-CD3/CD46 antibodies diluted in cold complete culture medium for 15 minutes on ice. The cells were then resuspended with 50 μL of warm complete culture medium containing F(ab’)2 rabbit anti mouse (7 μg/mL Jackson ImmunoResearch) and incubated for 2 minutes at 37°C. The reaction was stopped with cold complete culture medium. The cells were fixed with FoxP3/Transcription Factor Fixation and Permeabilization buffer (Thermo Fisher Scientific) for 50 minutes at 4°C and permeabilize with BD Perm buffer III (BD Bioscience) for 30 minutes at 4°C. Cells were stained with anti-phosphorylated Tyrosine FITC (p-Tyr clone PY20, Santa Cruz biotechnology, Cat# sc-508), anti-phosphorylated ERK APC (Thr202, Tyr204 clone MILAN8R, eBioscience, Cat# 14-9109-82) and anti-phosphorylated ZAP-70 APC (Y391, Y352 clone n3kpbu5, Invitrogen, Cat# 17-9006-42) diluted in Wash Buffer for 50 minutes at 4°C in the dark. The cells were analyzed the same day using a FortessaX20 (BD Biosciences). The level of phosphorylation was determined by calculating ΔMFI = MFI (CD3/CD46) – MFI (US – basal level off phosphorylation). Examples of staining are shown in Supplemental Figure 15.

### Confocal microscopy of activated CD4^+^ T cells over ICAM-1 or anti-CD3 and ICAM-1.

High-content screening 384-well imaging plates (CellCarrier-384 Ultra, Perkin Elmer) were coated overnight at 4°C with ICAM-1 (2 μg/mL, R&D Systems), for the experiment set-up of Figure 6. Alternatively, plates were coated with anti-CD3 (OKT3, 5 μg/mL, Biolegend) and ICAM-1 (2 μg/mL) overnight at 4°C for the experiment set-up of Supplemental Figure 4. The plates were then washed with PBS and cell culture medium before addition of preactivated CD4^+^ T cells by anti-CD3/CD46 for 5 days in the presence or absence of 1.25OH (9000 cells/well), followed by a 10-second centrifugation. After stimulation for 10 minutes at 37°C with ICAM-1 or 15 minutes with anti-CD3 and ICAM-1, the cells were fixed using 6% PFA. Non-adherent cells were removed by flipping the plate. Subsequently, the plate was washed with PBS and permeabilization buffer (PB, eBioscience), and then stained with Alexa Fluor 488 Phalloidin (Thermo Fisher Scientific), anti-pTyrosine antibody (PY99, Santa Cruz Biotechnology, Cat# sc-7020) or VDR-AF 647 (D-6, Santa Cruz biotechnology, Cat# sc-13133). The plate was incubated overnight at 4°C. The plate was then washed with PBS and stained with anti-Rabbit Alexa546 or anti-mouse IgG2b Alexa 546 antibodies (ThermoFisher Scientific) and DAPI (ThermoFisher Scientific). After 1 hour incubation, the plate was washed 3 times with PBS and subjected to automated confocal imaging. For the experiment in Figure 6, images were acquired with a high-content screening system (Opera Phenix, PerkinElmer) with a 40x 1.1 NA Plan Apochromat water immersion objective. For each well, 61 to 81 fields-of-view and 3 Z-planes per field (with step of 0.5 μm, starting at the cell contact plane with the substrate) were acquired. The Harmony software was used to perform automated analysis by extracting parameters corresponding to the mean of cells per well. The actin profile measures evaluate cell segmentation and/or actin distribution, by segmenting the cell in 5 regions from the cell membrane (the most outside segment, actin profile 1) to the center of the cell/nucleus region (profile 5). For the experiment in Supplemental Figure 4, images were acquired with a 63x 40x 1.1 NA Plan Apochromat water immersion objective and 10 Z-planes per field. The VDR intensity was calculated using maximum projection of the Z-planes, on the corresponding region of the nucleus and the cytoplasm after cell segmentation.

### Quantification of mRNA by qPCR.

After 5 days activation, cells were harvested and lysed in RLT Buffer supplemented with β-mercaptoethanol. Total RNA was extracted using the RNeasy Mini Kit (Qiagen) according to the manufacturer’s instructions. RNA concentration and purity were assessed using a Nanodrop spectrophotometer and cDNA was synthesized from total RNA using the Super Script III First-Strand Synthesis System (Invitrogen). The relative expression of CYP24A1 was quantified by real-time quantitative qPCR (RT-qPCR) using a Roche Lightcycler with PowerUp SYBR green mastermix (ThermoFisher) using the following primer sequences: Forward primer, GATTTTCCGCATGAAGTTGGGT, Reverse primer, CCTTCCACGGTTTGATCTCCA. The ribosomal protein RPL13A (Forward primer: ACCGTCTCAAGGTGTTTGACG, Reverse primer: GTACTTCCAGCCAACCTCGTG) and GAPDH (Forward primer: TGGAATTTGCCATGGGTGGA, Reverse primer: AGGTCGGAGTCAACGGATTT) were used as endogenous controls for normalization. Relative gene expression was calculated using the 2^–ΔCt^ method after normalization to the endogenous references genes and is presented as relative mRNA expression.

### Statistics.

Statistical analysis was performed using GraphPad Prism (Version 11). Normality for every data set was assessed with Shapiro-Wilk test. The appropriate statistical test was chosen based on the analysis conducted, as specified in the Figure legends. All data points are shown in the Figures. Each dot represents one donor. Data are shown as mean ± SEM. *P* ≤ 0.05 was considered statistically significant. All analyses were 2-sided.

### Study approval.

The study was approved by the French South-West & Overseas ethical committee and was registered at the French Ministry of Higher Education and Research (DC-20152488). Experiments were performed in agreement with the guidelines of the Declaration of Helsinki. The BIONAT study was approved by the Comité de Protection des Personnes (CPP) Sud Ouest et Outre Mer II on 06/02/2009 and the NEUROBANK cohort (CRB - CHU Toulouse, France) by the CPP # DC2016-2804. Both MS projects were declared to the Commission Nationale Informatique et Libertés.

### Data availability.

Values for all data points in graphs are reported in the Supporting data values file. Data are available upon request.

## Author contributions

SK and AD designed, conducted experiments, acquired and analyzed data. TA, BC, CG, FB, LN, JL, and EM conducted experiments, acquired and analyzed data. VDE analyzed data; BP provided data. MM, JS, JD analyzed data. RL, AS, and FM discussed experiments and data. LD designed experiments and wrote the manuscript. ALA designed the study, analyzed data and wrote the manuscript. SK and AD share cofirst authorship, SK being the first as she initiated the study and AD did the revision.

## Conflict of interest

The authors have declared that no conflict of interest exists.

## Funding support

- Agence Nationale de la Recherche ANR-19-CE14-0043–01 (to ALA).
- ANR-10-INBS-04 (to INFINITy Inserm UMR1291 core facility connected to ‘Toulouse Réseau Imagerie’ network, member of the France-BioImaging national infrastructure).
- ANR-23-CE15-0008-01 RETENTION (to FM).
- Fondation FranceSEP (to ALA and the 4th year PhD studentship for SK and AD).
- CNRS/University of Toronto Joint PhD Program (to ALA with Shannon Dunn, University of Toronto, Toronto, Canada, for AD).

## Supplementary Material

Supplemental data

Supporting data values

## Figures and Tables

**Figure 1:**
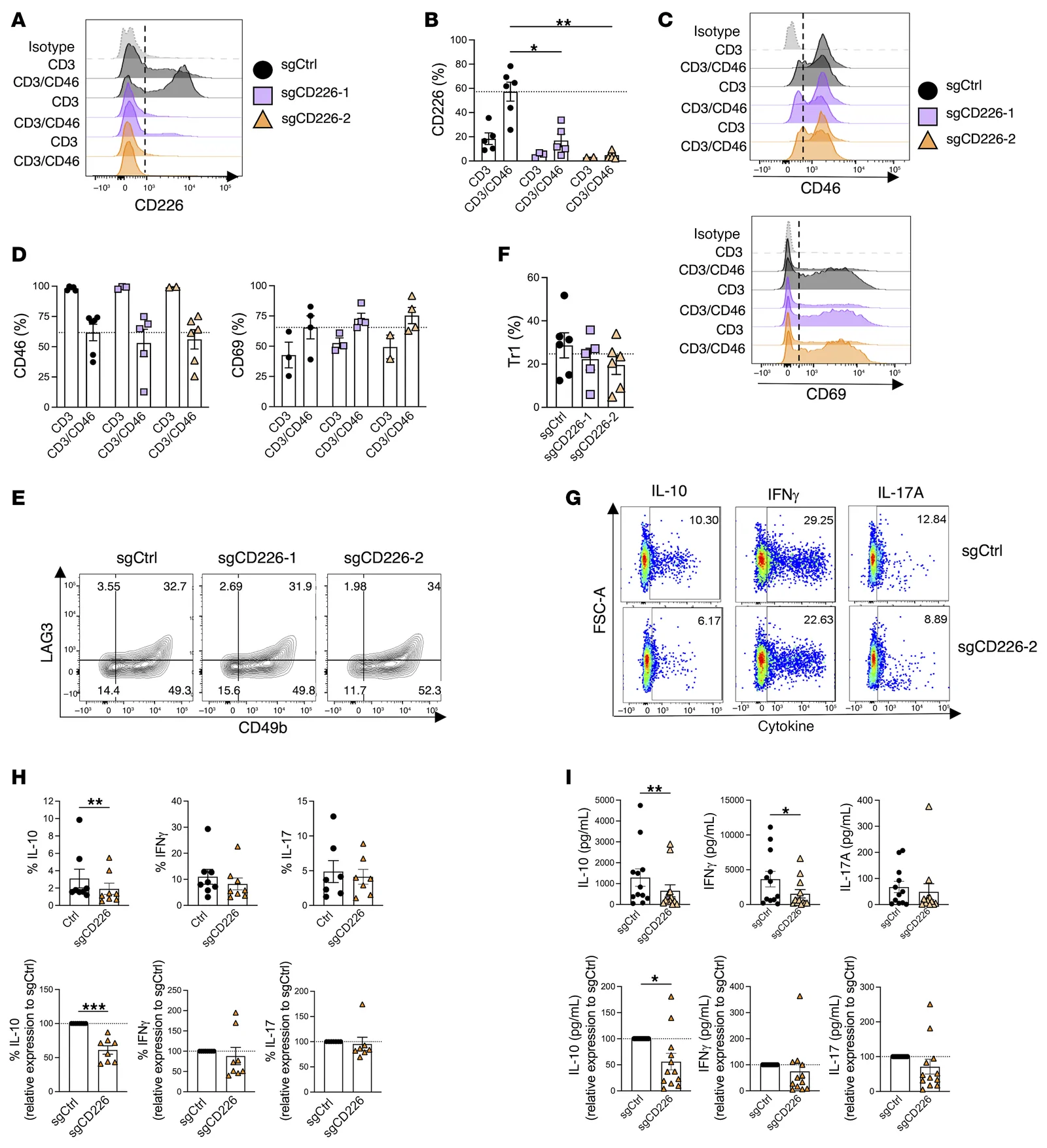
CD226 knockout by CRISPR/Cas9 impairs IL-10 production by CD46-activated primary CD4^+^ T cells. CD4^+^ T cells from HD homozygous for the CD226 rs763361 C/C genotype were nucleofected with either a control guide (sgCtrl) or 2 different guides directed against CD226 (sgCD226-1 and sgCD226-2), and then activated in vitro by anti-CD3 ± anti-CD46 antibodies. (**A**) Representative histograms and (**B**) data for all donors for CD226 expression (*n* = 2–6 donors according to the amounts of cells recovered). (**C**) Representative histogram and (**D**) data for all donors for CD46 and CD69 expression (*n* = 2–6 donors depending on the amounts of cells recovered). (**E** and **F**) Percentage of Tr1 based on CD49b and LAG3 coexpression after 5 days (*n* = 5 or 6 donors). (**G**) Representative dot plots and (**H**) data for all donors for intracellular production of IL-10, IFN-γ, and IL-17 by CD46-activated T cells for 3 days after transfection by sgCtrl or sgCD226-2 (*n* = 8 for each guide). (**I**) Secretion of IL-10, IFN-γ, and IL-17A was measured in culture supernatants by CBA in CD46-activated T cells for 5 days after transfection by sgCtrl or sgCD226-2 (*n* = 12 for each guide). For **H** and **I**, normalization was also done by calculating the relative change compared with cells with the sgCtrl (100%). Six independent experiments were performed that included sgCtrl and sgCD226-2. Mean ± SEM are shown. One-way ANOVA with Šídák’s post test (**B**, **D**, and **F**) or Wilcoxon *t* test (**H** and **I**).

**Figure 2:**
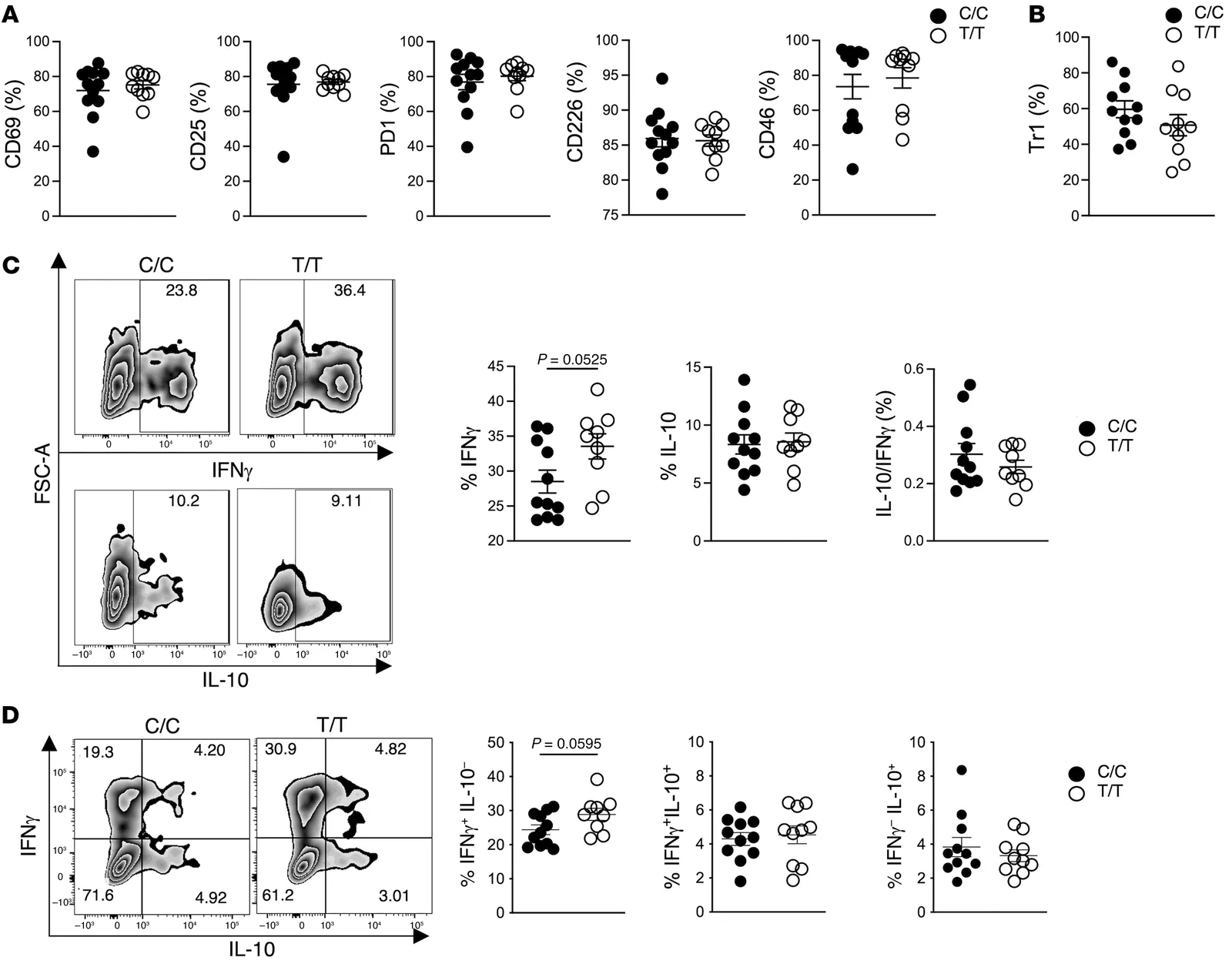
The *CD226* risk allele has no major impact on the CD46 pathway. Memory CD4^+^ T cells from HD homozygous for the CD226 rs763361 SNP (C/C, nonrisk or T/T, risk) were activated with anti-CD3/CD46 antibodies for 2 days. (**A**) Expression of CD69, CD25, PD1, CD226, and CD46 was determined by flow cytometry. (**B**) Percentage of Tr1 cells based on CD49b and LAG3 coexpression. Nonrisk (C/C, *n* = 10–12), risk (T/T, *n* = 10). (**C**) Representative dot-plots for IL-10 and IFN-γ secretion and data for all donors. (**D**) Proportion of IL-10– and IFN-γ–producing cells in the IL-10/IFN-γ quadrants. Nonrisk (C/C, *n* = 11), risk (T/T, *n* = 9). Unpaired *t* test, mean ± SEM. Ten independent experiments were performed that included pairs of donors expressing the *CD226* nonrisk or risk allele.

**Figure 3:**
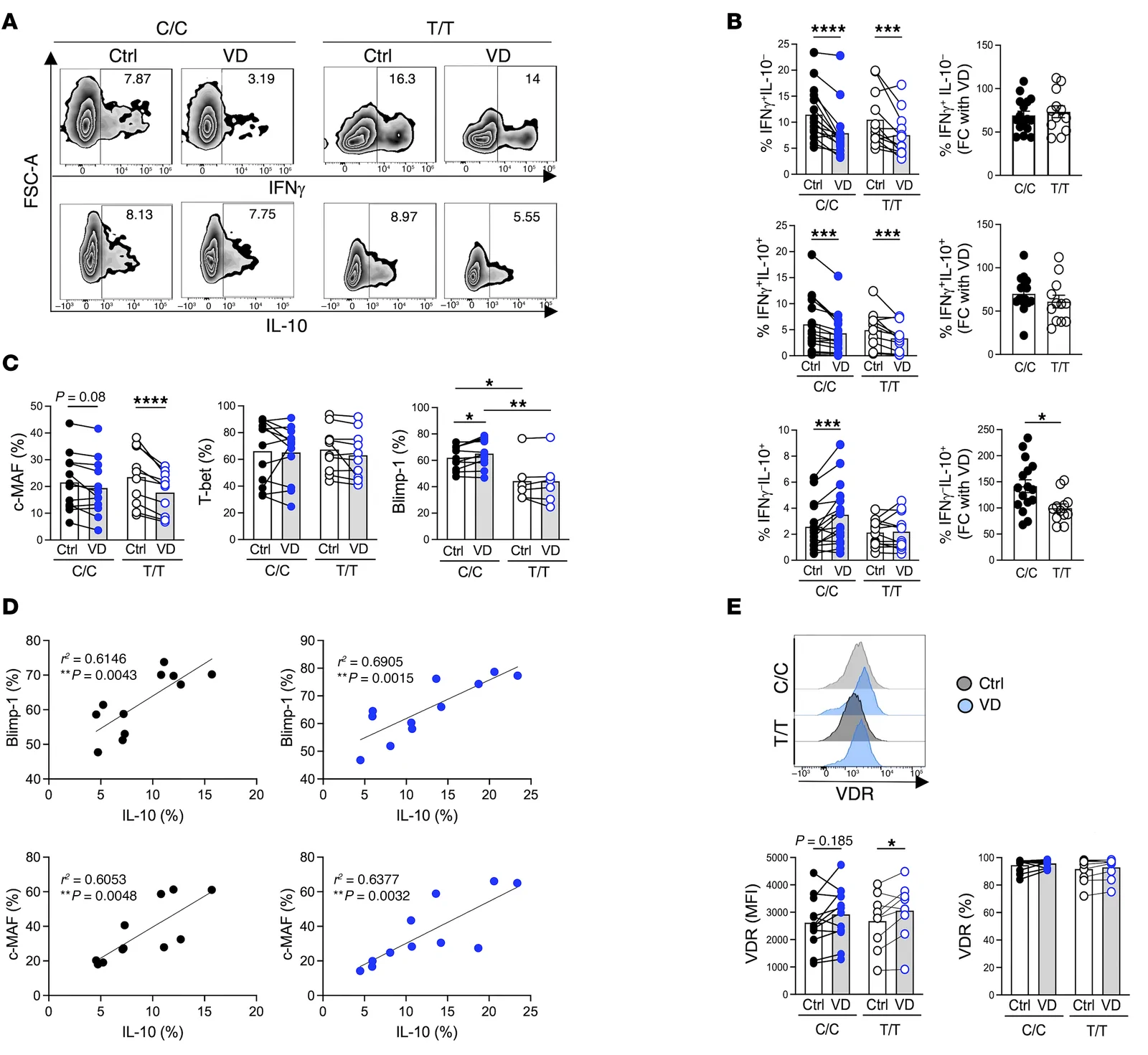
The *CD226* risk allele impairs response to VD. CD4^+^ T cells from HD homozygous for either of the CD226 rs763361 variants (C/C, nonrisk or T/T, risk), were activated with anti-CD3/CD46 antibodies with (VD) or without (Ctrl) 1,25OH. (**A**) Representative dot plots for IL-10 and IFN-γ intracellular production after 2 days activation. (**B**) Proportion of IL-10– and IFN-γ–producing cells in the IL-10/IFN-γ quadrants for all donors, and relative fold change with VD compared with the control. C/C donors (*n* = 16) and T/T donors (*n* = 12). (**C**) c-MAF, T-bet and Blimp-1 expression was assessed by flow cytometry (C/C donors (*n* = 14, and 11 for Blimp-1) and T/T donors (*n* = 10 and 6 for Blimp-1)). (**D**) Correlation (Pearson’s) in HD homozygous for the C/C allele between expression of IL-10 and Blimp-1 (upper panels) and c-Maf (lower panels) in the absence (black circles, left panels) or the presence of VD (blue circles, right panels). *n* = 11 donors. (**E**) Expression of VDR assessed by flow cytometry at D5. Nonrisk (C/C, *n* = 11), risk (T/T, *n* = 10). One-way ANOVA with Šídák’s post test (**B**, **C**, and **E**) or Mann-Whitney *t* test (**B**, for relative fold change), mean ± SEM are shown. At least 4 independent experiments were performed that included both C/C and T/T individuals.

**Figure 4:**
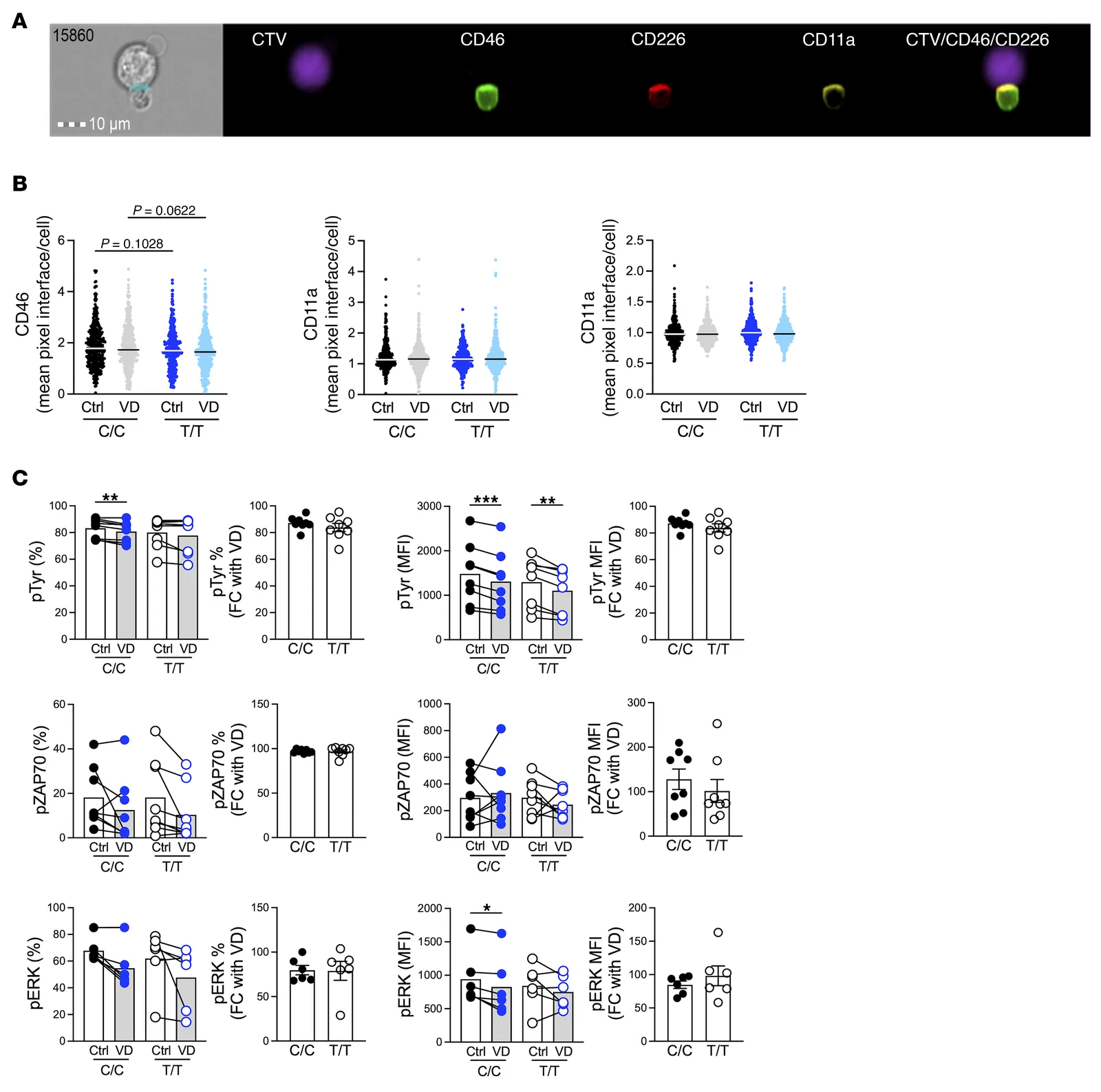
The *CD226* risk allele does not modify early signaling events in the presence of VD. CD4^+^ T cells from HD homozygous for the *CD226* rs763361 SNP (C/C, nonrisk; T/T, risk) preincubated with 1,25OH were cocultured with anti-CD3/CD46 antibody-precoated CTV-labelled P815 cells, used as a model APC, for 15 minutes. The conjugates were labeled as indicated and acquired at original magnification × 40 using the ImageStream Mark II Amnis flow cytometer. Recruitment at the immune synapse (IS) was quantified by applying a mask at the IS and calculating the median pixel intensity interface/whole cell. (**A**) Representative image of the staining obtained by the flow imager at the interface between the APC-T cell. (**B**) Plots showing recruitment of CD46, CD226, and LFA-1 at the IS. Four independent experiments were performed that included both the nonrisk and risk allele. Nonrisk (C/C, *n* = 4), risk (T/T, *n* = 4) with a maximum of 100 conjugates per donor. Mean ± SEM, 1-way ANOVA with Šídák’s post test. (**C**) CD4^+^ T cells preincubated with 1,25OH overnight were activated for 2 minutes by anti-CD3/CD46 antibodies and phosphorylation of tyrosine (p-Tyr), ZAP70 (p-ZAP70) and ERK (p-ERK) assessed by flow cytometry (*n* = 6–8, 1-way ANOVA paired test with Šídák’s post test). The fold change in phosphorylation upon addition of VD is also shown (Mann-Whitney *t* test). Three independent experiments were performed that included both the nonrisk and risk allele.

**Figure 5:**
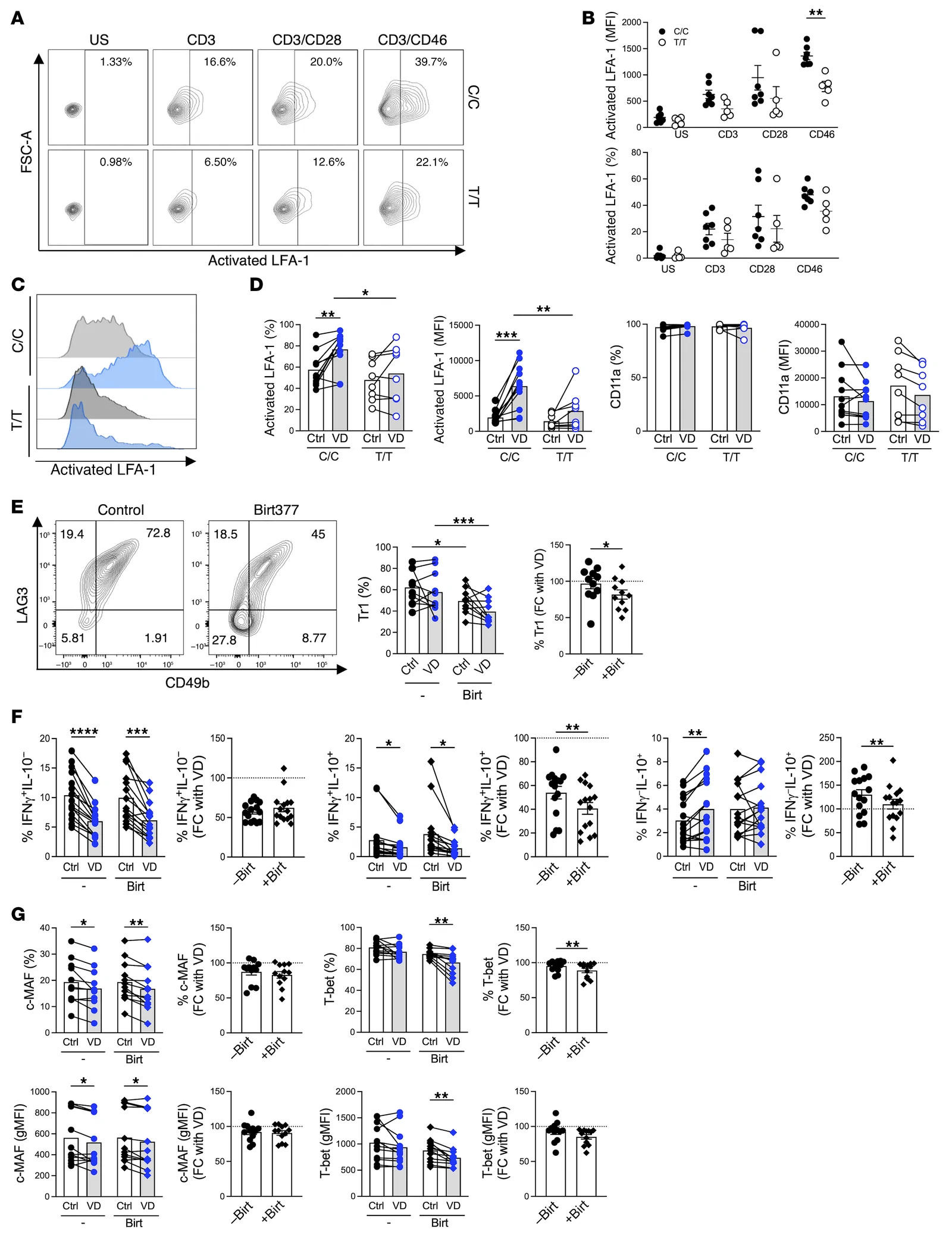
The *CD226* risk allele reduces activation of LFA-1 in the presence of VD that is required for IL-10 production. (**A**) CD4^+^ T cells from HD homozygous for the *CD226* rs763361 SNP (C/C, Nonrisk; T/T, risk) were activated with anti-CD3, anti-CD3/CD28, or anti-CD3/CD46 antibodies for 5 days. (**A**) Expression of activated LFA-1. (**B**) Dot plot showing all donors. *n* = 7 C/C and 5 T/T; mean ± SEM, 5 independent experiments that included both nonrisk and risk allele carrying individuals, 1-way ANOVA with Šídák’s post test. (**C**) CD4^+^ T cells from HD homozygous for the *CD226* rs763361 SNP (C/C, nonrisk; T/T, risk) were activated with anti-CD3/CD46 with (VD) or without (Ctrl) 1,25OH for 5 days. Representative expression of activated LFA-1. (**D**) Dot plot showing expression of activated LFA-1 and CD11a for all donors (*n* = 11 C/C and 8 T/T, 5 independent experiments that included both nonrisk and risk allele). One-way ANOVA with Šídák’s post test. (**E**) CD4^+^ T cells from HD homozygous for the *CD226* rs763361 C/C genotype were activated with anti-CD3/CD46 antibodies for 5 days in the presence or absence of 1,25OH and with or without BIRT377 (Birt), an antagonist of LFA-1 activation. One representative dot plot for Tr1 cell identification and quantification for *n* = 11 donors each, and the relative change in the presence of VD compared with the control is also calculated. Four independent experiments were performed. (**F**) Proportion of IL-10– and IFN-γ–producing cells in the IL-10/IFN-γ quadrants for all donors, and the percentage of change with VD compared with the control (*n* = 15 donors). (**G**) c-MAF and T-bet expression was assessed by flow cytometry (*n* = 12). One-way ANOVA with Šídák’s post test or paired *t* test for relative fold change. Nine independent experiments were performed that included both nonrisk and risk allele.

**Figure 6:**
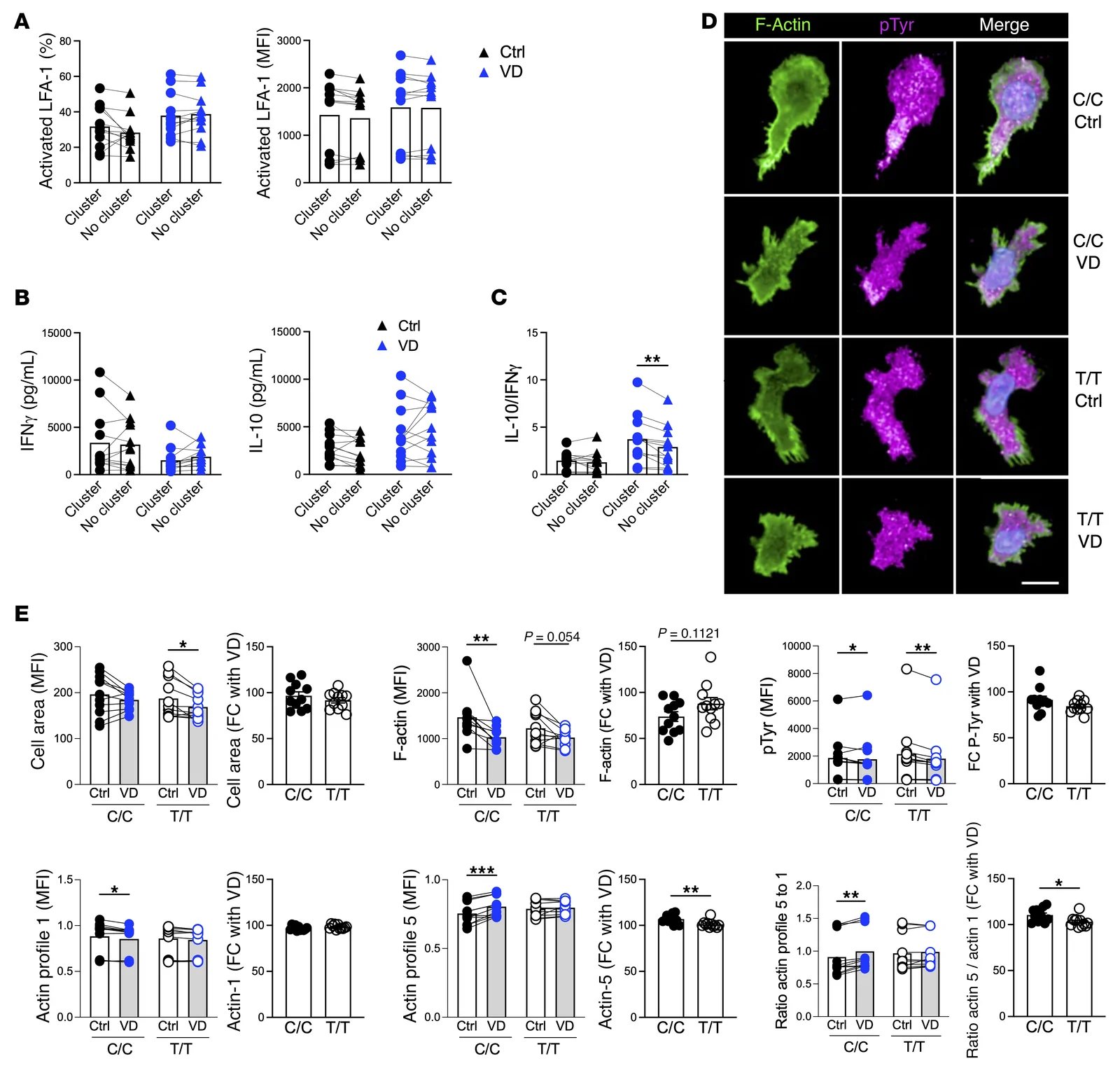
LFA-1 mediated–homotypic interactions are required for optimal IL-10 production. CD4^+^ T cells from HD homozygous for the C/C CD226 rs763361 SNP (nonrisk) were activated by anti-CD3/CD46 antibodies in the presence of 1,25OH and the clusters were disrupted by gentle pipetting twice a day. (**A**) LFA1 activation was assessed by flow cytometry and (**B**) production of IL-10 and IFN-γ in the supernatants was assessed by CBA after 5 days. (**C**) The IL-10/IFN-γ ratio is shown, 1-way ANOVA with Šídák’s post test, *n* = 12, 5 independent experiments. (**D**) CD4^+^ T cells from HD homozygous for either of the *CD226* rs763361 SNP (C/C, nonrisk; T/T, risk) were activated with anti-CD3/CD46 antibodies for 5 days in the presence or absence of 1,25OH, and then plated on precoated ICAM-1 for 10 minutes before fixation and staining for F-actin, and phosphorylated Tyr (pTyr) and automated confocal imaging and quantification were performed. Representative images are shown. (**E**) Quantified data obtained for the 11 donors each. Four independent experiments were performed that included both nonrisk and risk allele. One-way ANOVA with Šídák’s post test or Welch’s test for relative fold change.

**Figure 7:**
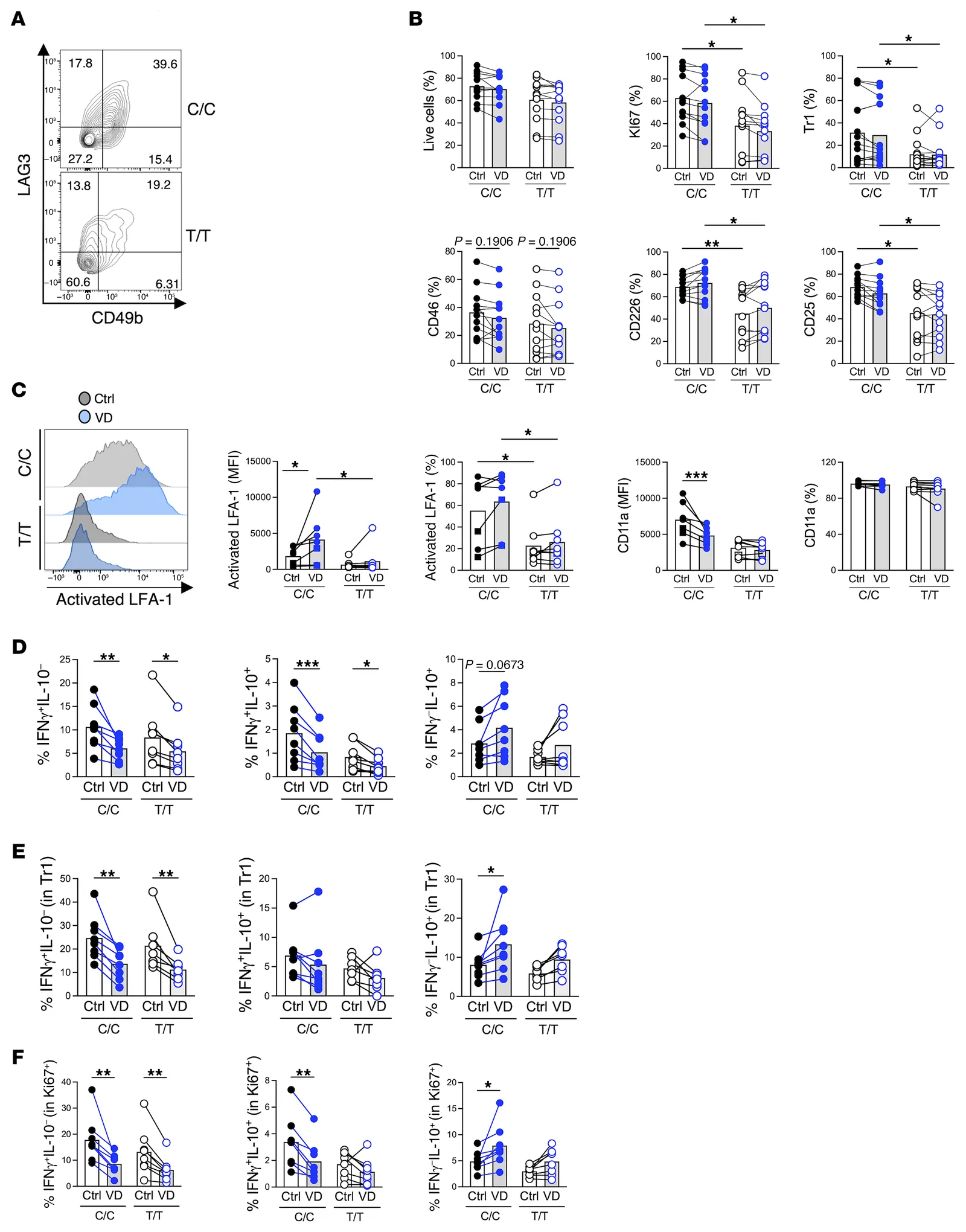
The *CD226* risk variant reduces activation of LFA-1 in MS T cells. CD4^+^ T cells from patients with MS homozygous for the *CD226* rs763361 C/C or T/T genotype, were activated with anti-CD3/CD46 for 5 days. (**A**) Representative Tr1 staining. (**B**) The percentage of live cells, Ki67^+^ and Tr1 cells, as well as expression of CD25, CD226 and CD46 is represented for both genotypes (*n* = 15 C/C and 15 T/T, mean ± SEM, 6 independent experiments were performed that included both nonrisk and risk allele). (**C**) Representative histograms of the effect of VD on the expression of activated LFA-1 assessed by flow cytometry, and data for all donors (*n* = 7 C/C and 7 T/T, mean ± SEM, 4 independent experiments were performed that included both nonrisk and risk allele). (**D**) The proportions of IL-10- and IFN-γ–producing CD4^+^ T cells in the IL-10/IFN-γ quadrants were analyzed by flow cytometry. (**E**) Proportion of Tr1 cells producing IL-10 and IFN-γ in the IL-10/IFN-γ quadrants. (**F**) Proportion of Ki67^+^ CD4^+^ T cells producing IL-10 and IFN-γ in the IL-10/IFN-γ quadrants. *n* = 8 C/C and 8 T/T (7 T/T for the Tr1 and Ki67 gates as limited cell numbers for 1 donor) performed in 4 independent experiments. One-way ANOVA with Šídák’s post test.

**Table 2:**
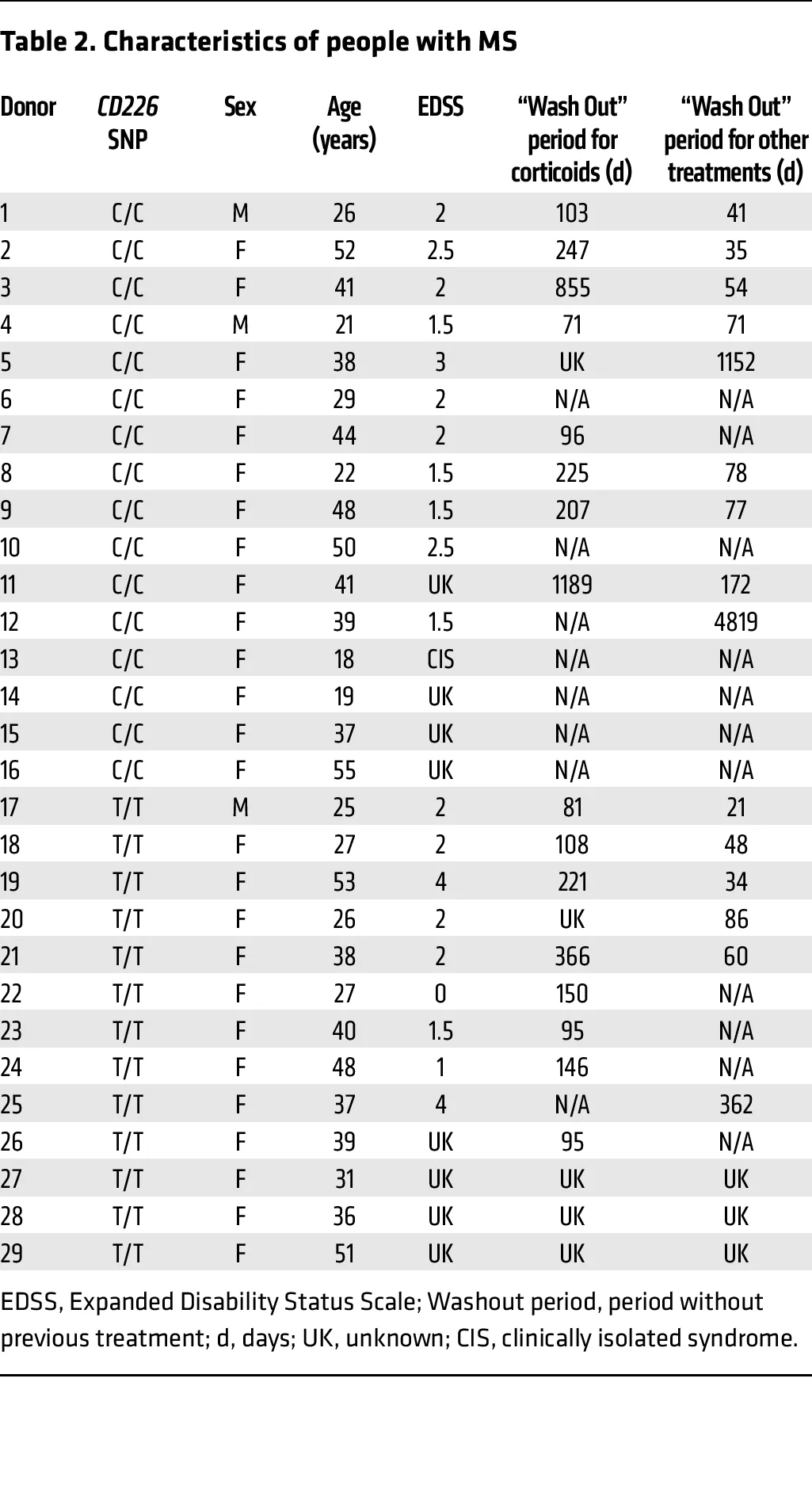
Characteristics of people with MS

**Table 1:**
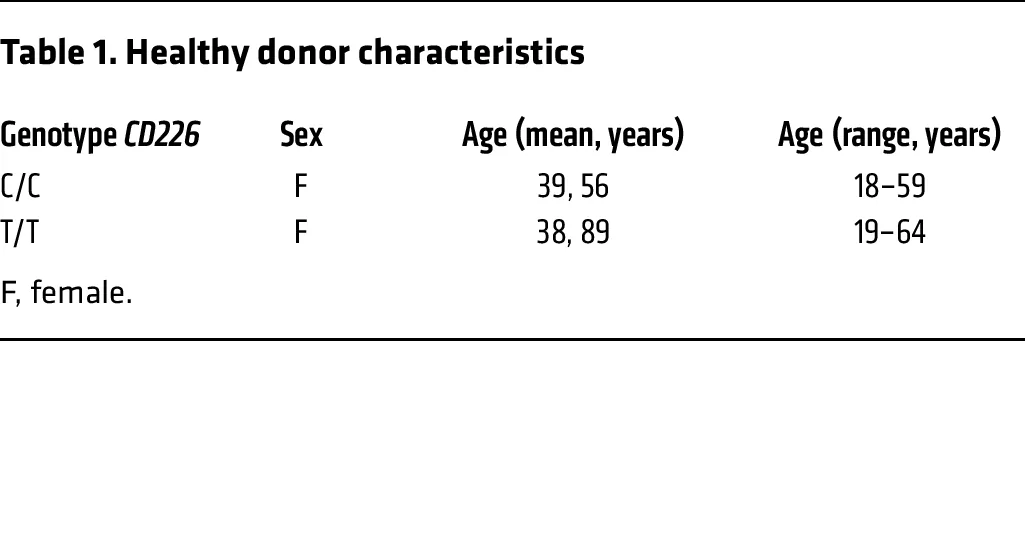
Healthy donor characteristics

## Version 1 09/01/2026

In-Press Preview

## Version 2 10/15/2026

Electronic publication
