# Supplementary material for: Multiple sclerosis–associated CD226 variant disrupts vitamin D–induced type I Treg differentiation by impairing LFA-1 activation
Source: J Clin Invest. 2026 Sep 1;136(20):e202193. doi: 10.1172/JCI202193 (PMC13668641; doi:10.1172/JCI202193)
Supplement: Supplementary file 1 [file jci-136-202193-s235.pdf]

Multiple sclerosis-associated CD226 variant disrupts vitamin D-induced type I Treg differentiation by impairing LFA-1 activation

Supplemental Figures – Kari *et al*

Supplemental Figure 1

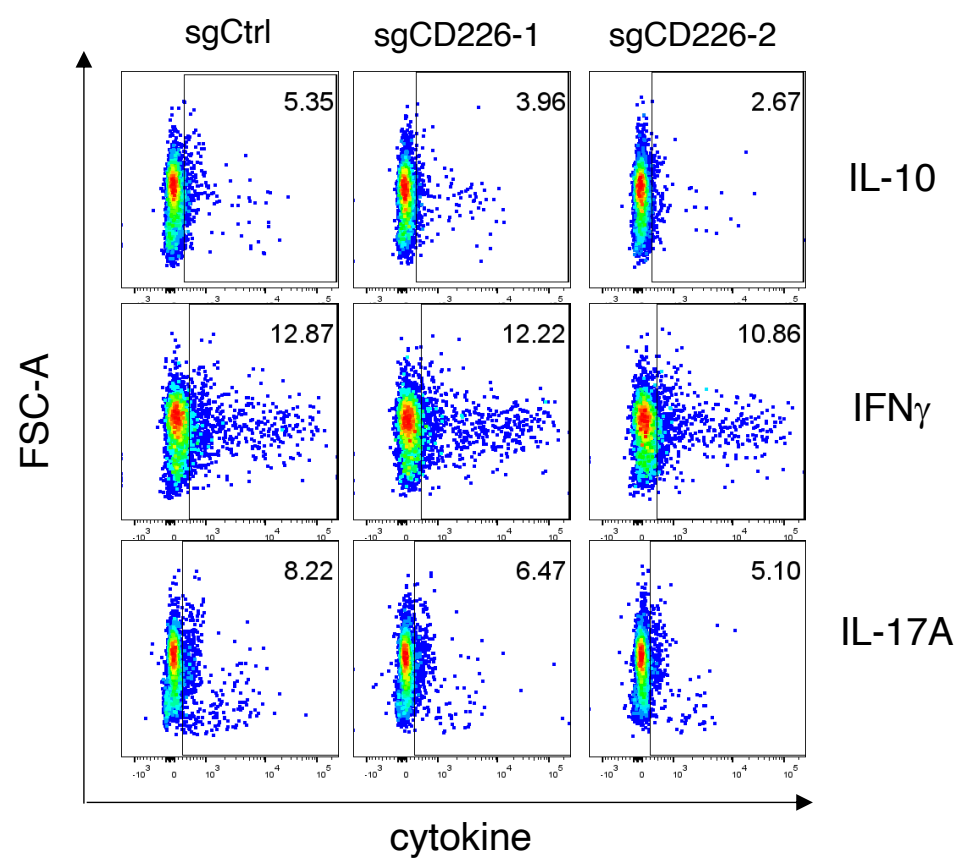

Supplementary Fig. 1. Cytokine production profile upon CD226 deletion. $CD4^+$ T cells from HD homozygous for the *CD226* rs763361 C/C genotype were nucleofected with either a control guide (sgCtrl) or two different guides directed against CD226 (sgCD226-1 and sgCD226-2), and then activated *in vitro* by anti-CD3/anti-CD46 antibodies for 2 days. Representative flow cytometry plot showing the production of IL-10, IFNγ and IL-17A. Representative of three donors.

Supplemental Figure 2

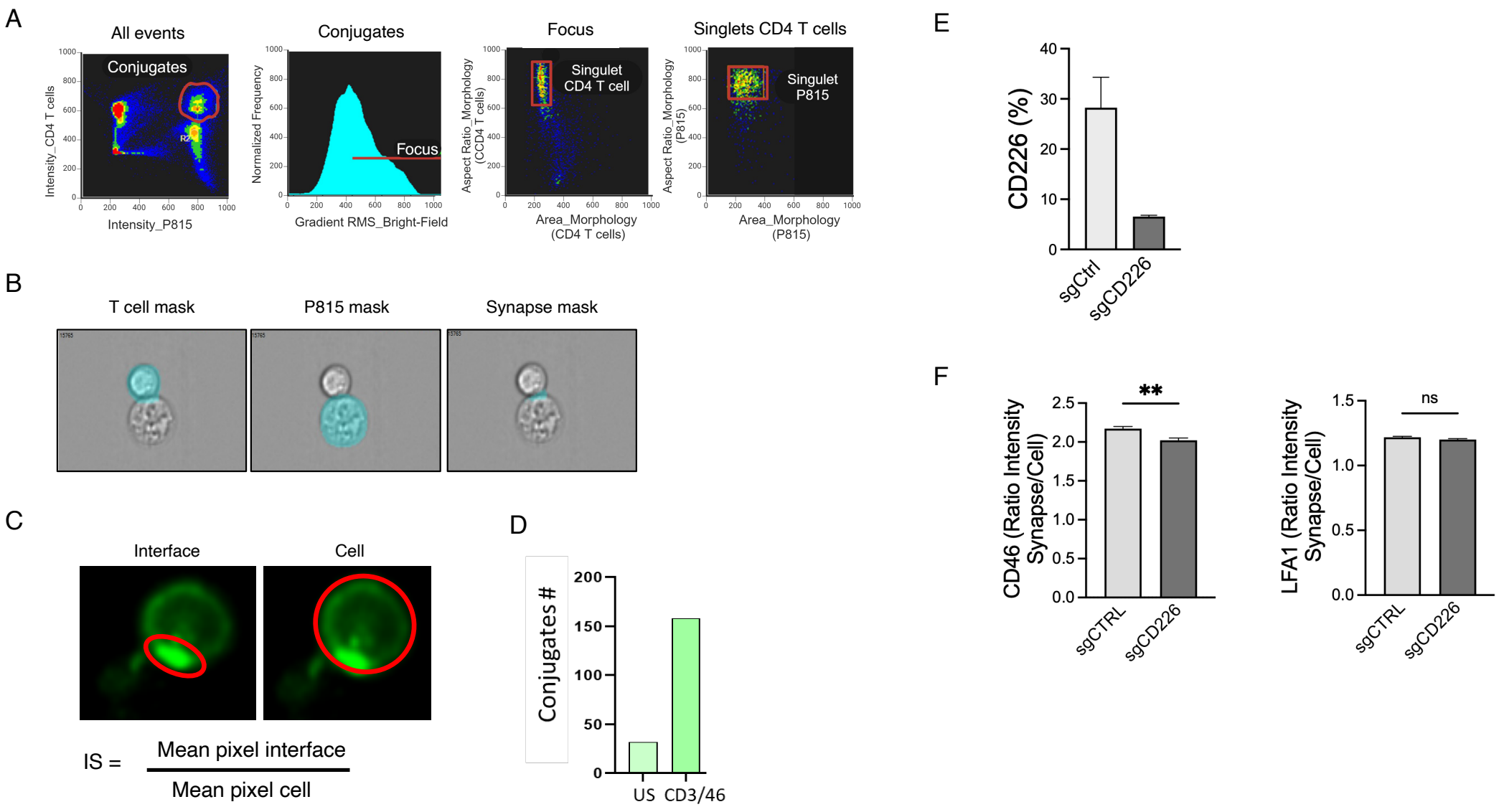

Supplemental Figure 2. Analysis of the recruitment of proteins at the immune synapse (IS). (A) CD4^{+} T cells were co-cultured with anti-CD3/CD46-pre-coated CTV-labelled P815 cells, used as a model APC, for 15 min. Cells were labeled as indicated and acquired by imaging flow cytometer. The strategy to select conjugates in focus with 1 CD4/1 P815 is indicated. **(B)** Masks on the T cells, on the P815, and at the IS required to quantify recruitment at the IS. **(C)** Recruitment is determined by calculating the ratio median pixel intensity interface / whole T cell. **(D)** Numbers of conjugates obtained between T cells and uncoated P815 (US) or CD3/CD46-coated P815. **(E)** CD4^{+} T cells from HD homozygous for the *CD226* rs763361 C/C genotype were nucleofected with either a control guide (sgCtrl) or a guide directed against CD226 (sgCD226-2), and then activated *in vitro* by anti-CD3/anti-CD46 antibodies. CD226 expression was assessed by flow cytometry after 2 d. **(F)** The recruitment of CD46 and CD11a was assessed at the IS by forming conjugates between T cells and anti-CD3/CD46 antibodies-pre-coated CTV-labelled P815 cells, used as a model APC, for 15 min. The conjugates were labeled as indicated and acquired at 40X using the ImageStream Mark II Amnis flow cytometer. Recruitment at the IS was quantified by applying a mask at the IS and calculating the median pixel intensity interface / whole cell. Mann-Whitney t-test.

Supplemental Figure 3

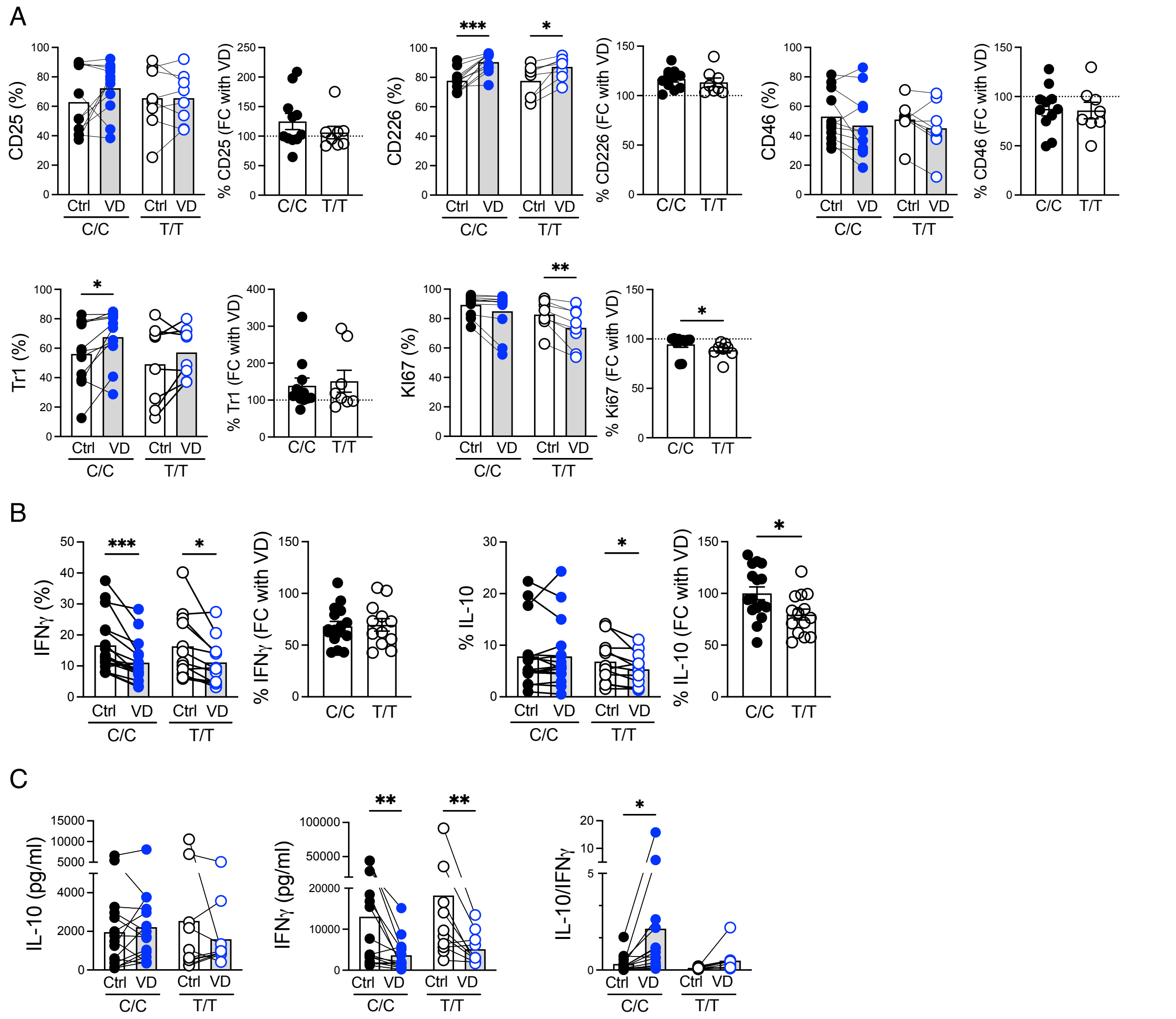

Supplemental Figure 3. Impact of the *CD226* risk allele on T cell phenotype in response to VD. $CD4^+$ T cells from HD homozygous for the *CD226* rs763361 variant, C/C, non-risk or T/T, risk, were activated with anti-CD3/CD46 with or without 1,25OH for 5 d. (**A**) Expression of CD25, CD226, CD46 and Ki67, as well as the percentage of Tr1 cells was assessed by flow cytometry, and the percentage of change with VD was calculated. Mean ± SEM: C/C, n=11, T/T, n=8. One-way ANOVA with Sidak's post-test and Mann-Whitney t-test for relative fold change. (**B**) Expression of IFNγ and IL-10 was assessed by flow cytometry, and the relative fold change with VD compared to the control is shown. Mean: C/C, n=17, T/T, n=12. One-way ANOVA with Sidak's post-test and Welch's test. (**C**) Secretion of IL-10 and IFNγ in the culture supernatants was measured by CBA. The ratio IL-10/ IFNγ is also shown. Mean: C/C, n=15, T/T, n=10. One-way ANOVA with Sidak's post-test.

Supplemental Figure 4

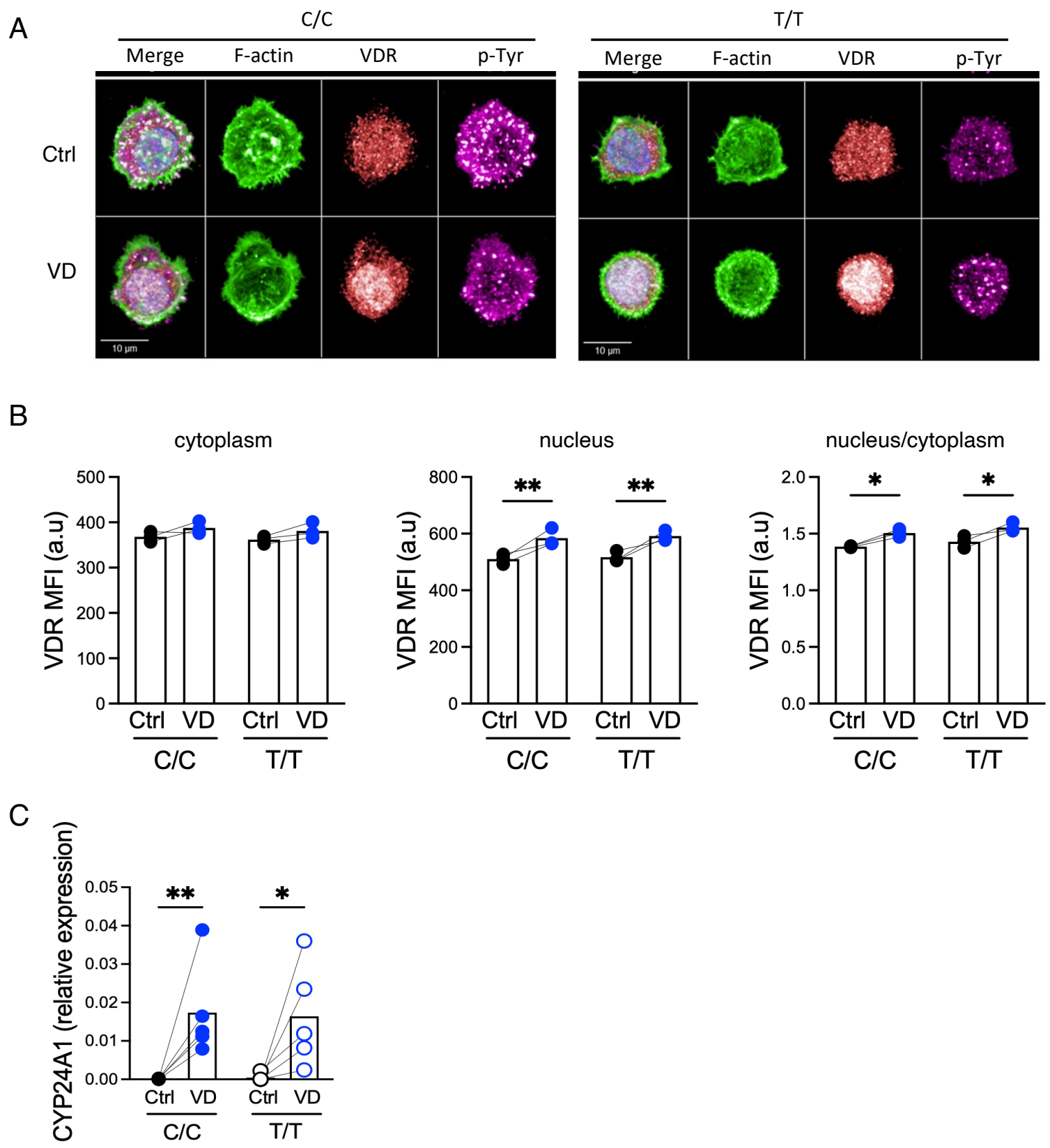

Supplemental Figure 4. No difference in VDR translocation and CYP24A1 expression between the two genotypes. (A) CD4+ T cells from HD homozygous for either of the CD226 rs763361 SNP (C/C, non-risk; T/T, risk) were activated with anti-CD3/CD46 antibodies for 2 d in the presence or absence of 1,25OH, and then plated on precoated anti-CD3 and ICAM-1 for 15 min before fixation and staining for F-actin and VDR. Automated confocal imaging and quantification were performed. Representative images are shown. **(B)** The VDR mean fluorescence intensity calculated on the cytoplasm (left), the nucleus (middle) and the ratio nucleus/cytoplasm (right) is represented. Two independent experiments were performed that included both protective and risk allele (C/C, non-risk; T/T, risk; n=3 each). (**C**) Relative expression of CYP24A1 was measured by RT-qPCR on CD4+ T cells activated by CD3/CD46 for 5 d (n= 5 C/C and 5 T/T). One-way ANOVA with Sidak's post-test.

Supplemental Figure 5

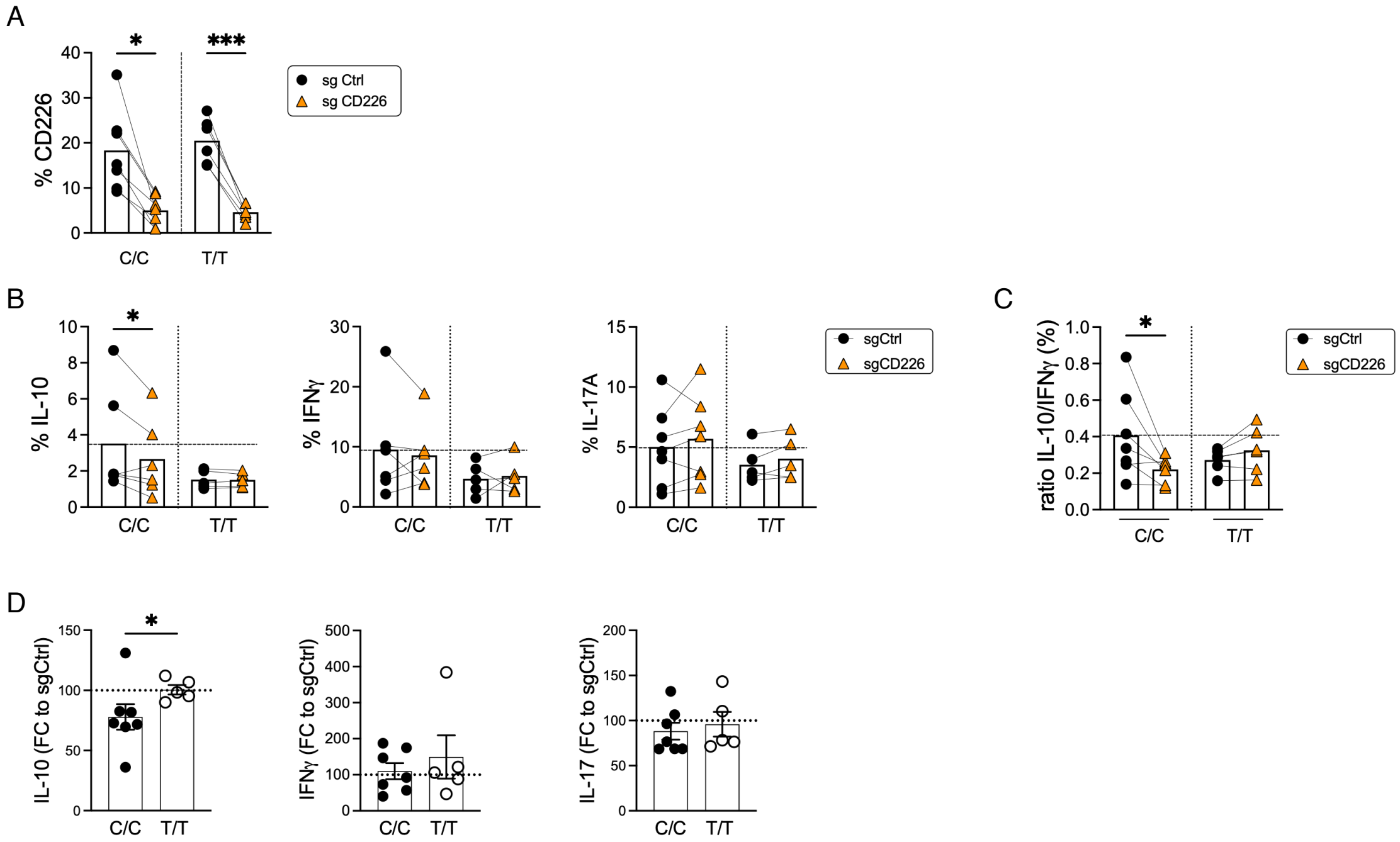

Supplemental Figure 5. Effect of CD226 deletion on cytokine production of CD4+ T cells from C/C and T/T donors. $CD4^+$ T cells from HD either homozygous for the *CD226* rs763361 C/C and T/T genotype were nucleofected with either a control guide (sgCtrl) or the sgCD226-2. Cells were activated *in vitro* by anti-CD3/- anti-CD46 antibodies for 2 d. (**A**) Expression of CD226 in C/C and T/T donors. (**B**) Effect of CD226 deletion on production of IL-10, IFNγ and IL-17A. (**C**) The ratio IL-10/IFNγ was calculated. (**D**) The percentage of control values was calculated to compared the effect of CD226 deletion in both genotypes. (n= 7 C/C and 5 T/T). One-way ANOVA with Sidak's post-test (A, B, C) or unpaired t-test (D).

Supplemental Figure 6

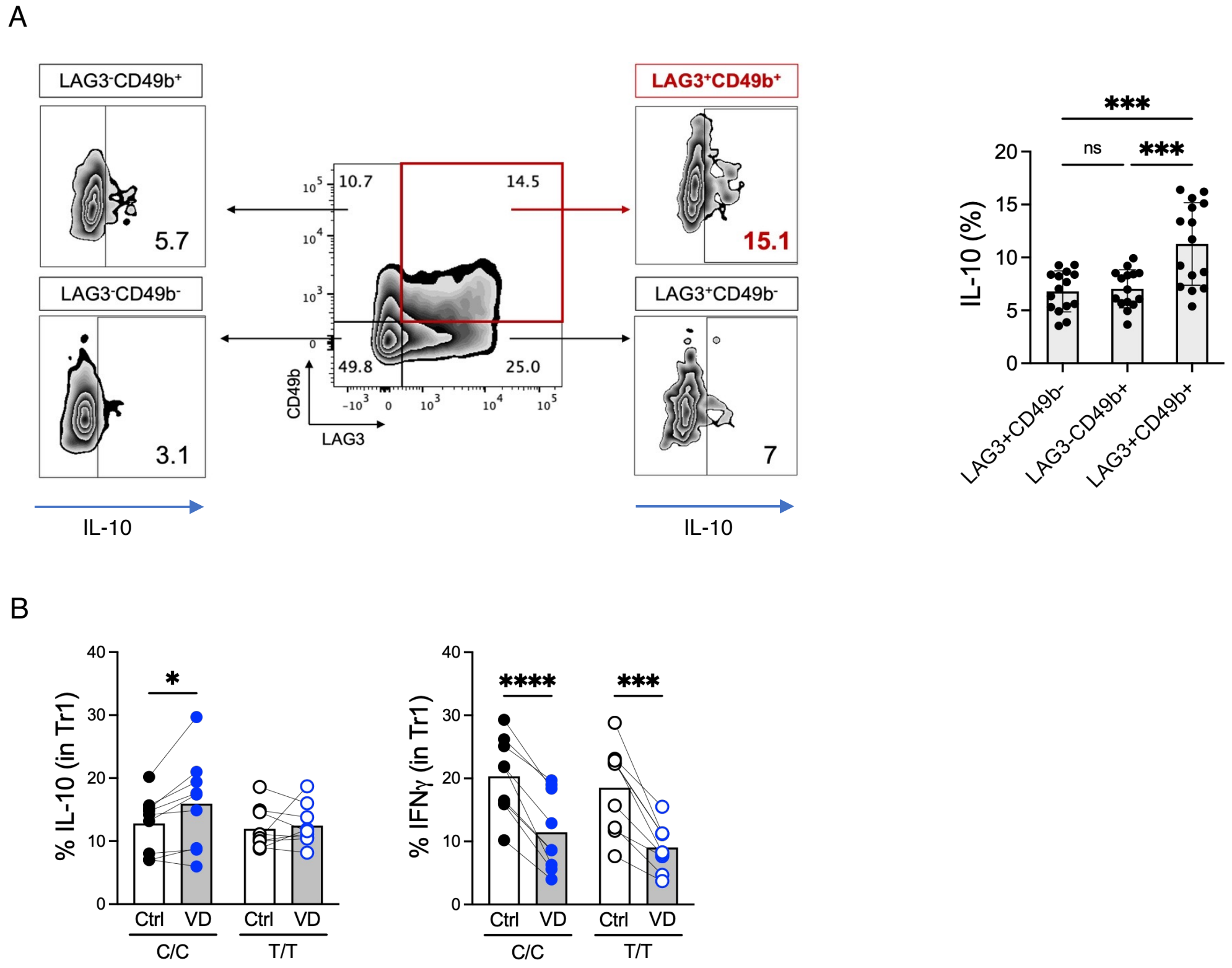

Supplemental Figure 6. 1,25OH promotes IL-10 production by Tr1 cells only in C/C donors. (**A**) Representative flow cytometry plots from one donor and pooled quantitative data showing IL-10 production by CD4^{+} T cells within each LAG/CD49b quadrant after 2 d of CD3/CD46 stimulation. The LAG3^{+}CD49b^{+} population corresponds to the Tr1 cells. n=15, Mean ± SEM. One-way ANOVA with Sidak's post-test (**B**) CD4^{+} T cells from HD homozygous for the CD226 rs763361 C/C or T/T genotypes were activated *in vitro* by anti-CD3 and anti-CD46 antibodies for 2 d in the absence or presence of 1,25OH. IL-10 and IFNγ production by Tr1 were analyzed. (n= 9 C/C and 9 T/T). One-way ANOVA with Sidak's post-test.

Supplemental Figure 7

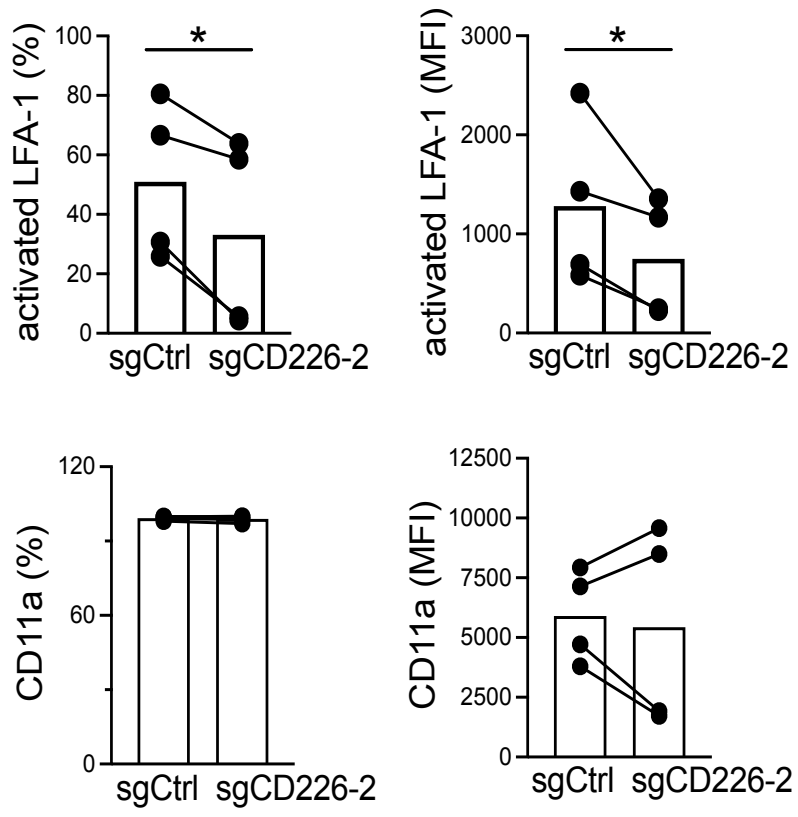

Supplemental Figure 7. Deletion of CD226 reduces activation of LFA-1. Expression of activated LFA-1 and CD11a in CD3/CD46-costimulated CD4 T cells deleted in *CD226* by CRISPR/Cas9 (sgCD226-2) was determined by flow cytometry. n=4 donors, paired t-test.

Supplemental Figure 8

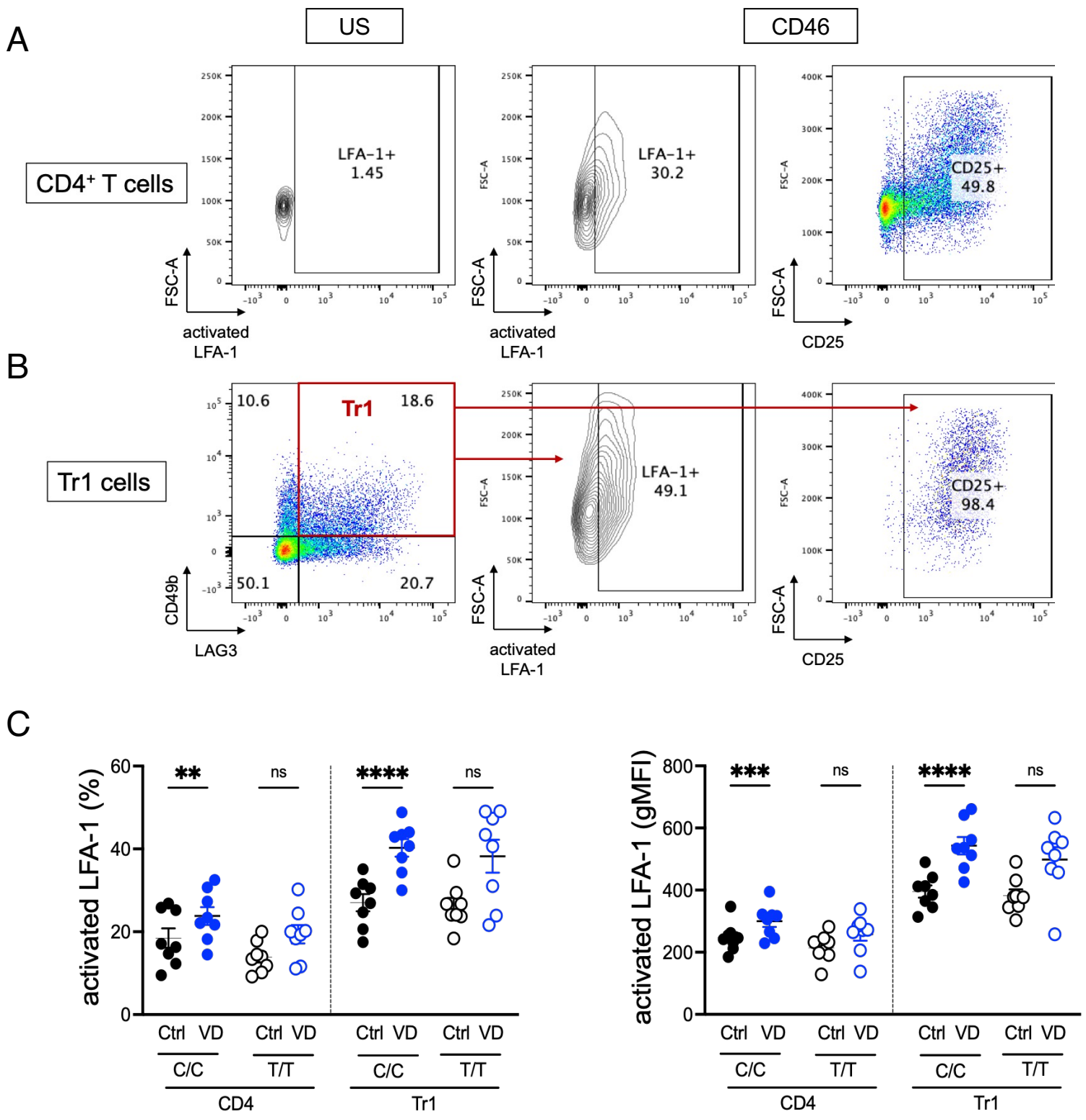

Supplemental Figure 8. 1,25OH promotes LFA-1 activation in Tr1 cells. CD4+ T cells from HD homozygous for the CD226 rs763361 C/C or T/T genotypes were activated *in vitro* by anti-CD3 and anti-CD46 antibodies for 2 d in the absence or presence of 1,25OH. LFA-1 activation was analyzed by flow cytometry in CD4+ T cells and in the Tr1 population. **(A)** Representative flow cytometry plots from one C/C donor showing LFA-1 activation in CD4+ T cells in unstimulated cells and LFA-1 activation and CD25 expression in CD3/CD46 activated cells. **(B)** Tr1 cell identification and analysis of activated LFA-1 and CD25 expression within the Tr1 population. **(C)** Expression of LFA-1 activation in the absence or presence of 1,25OH in CD4+ T cells and Tr1 cells from C/C and T/T donors. n= 8 donors from each genotype. Mean ± SEM, one way ANOVA with Sidak's post-test.

Supplemental Figure 9

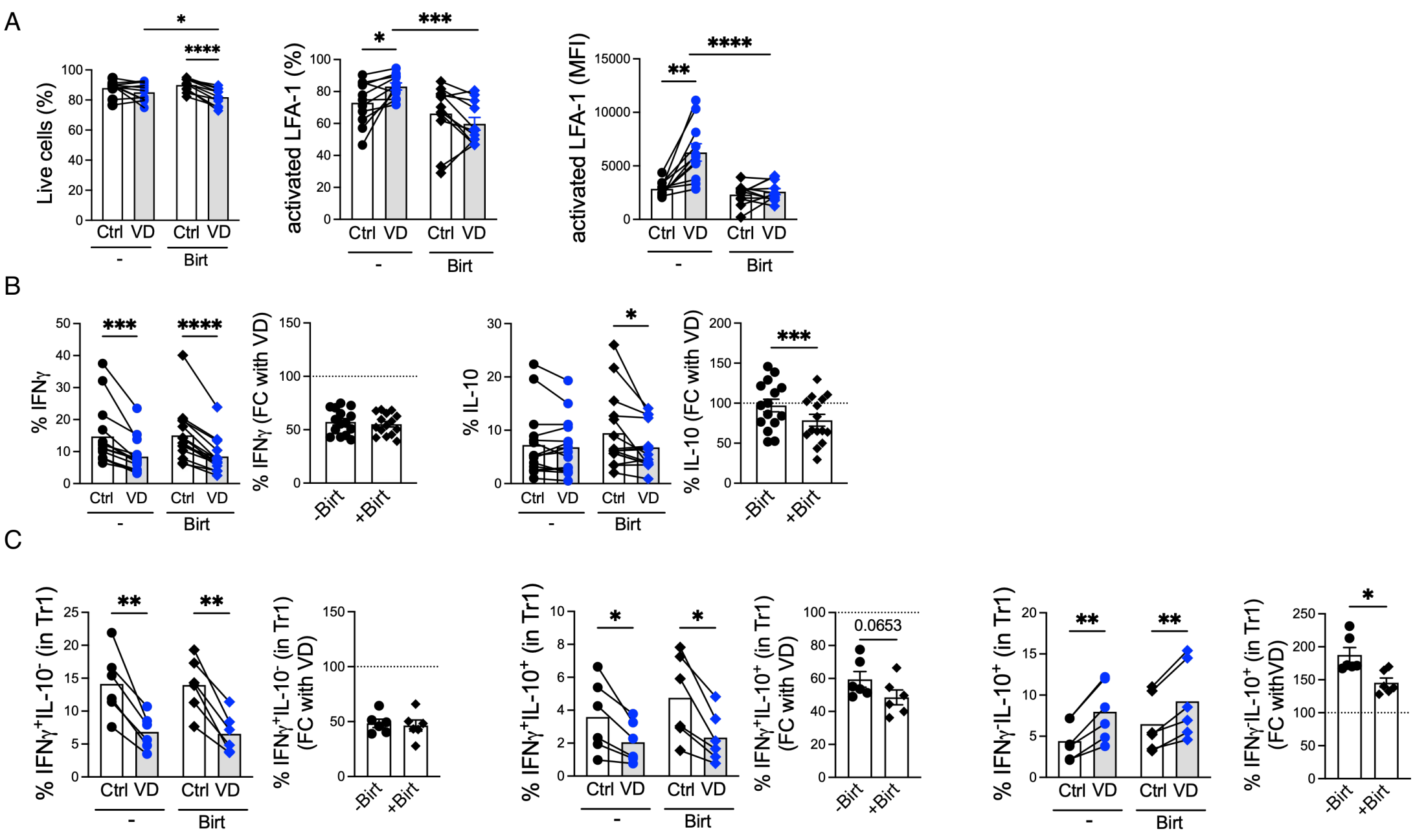

Supplemental Fig. 9. Phenotype and cytokine production of CD4⁺ T cells activated in the presence of an LFA-1 allosteric antagonist. CD4⁺ T cells from HD homozygous for the *CD226* rs763361 C/C allele were activated with anti-CD3/CD46 for 5 d with (VD) or without (Ctrl) 1,25OH, and with (Birt) or without (-) BIRT377, an antagonist of LFA-1 activation. (**A**) Percentage of live cells and expression of activated LFA-1. Mean, n=11, one way ANOVA with Sidak's post-test. (**B**) IFNγ and IL-10 production by CD4⁺ T cells was analyzed by intracellular staining, and the percentage of change with VD was calculated. Nine independent experiments were performed (n=15). (**C**) The effect of BIRT377 on IL-10/IFNγ expression was assessed within the Tr1 population, and the percentage of change induced by VD treatment was calculated. Two independent experiments (n=6). One way ANOVA with Sidak's post-test, and Mann-Whitney t-test (for relative fold change), mean ± SEM.

Supplemental Figure 10

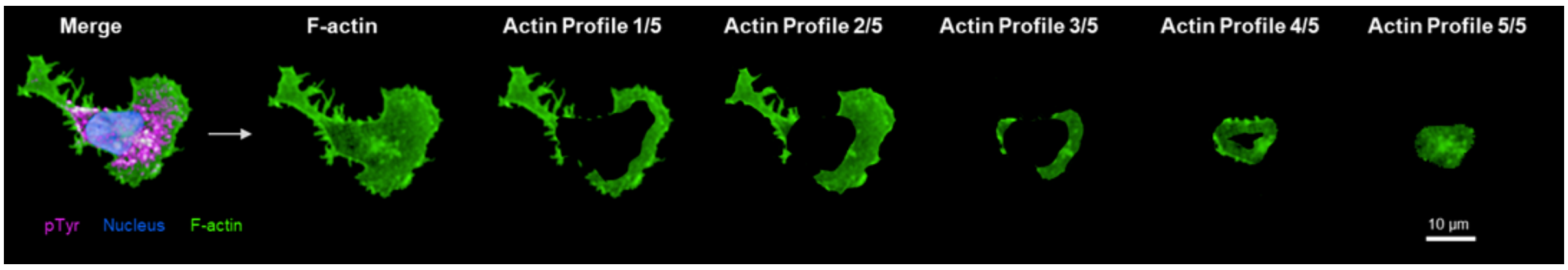

Supplementary Figure 10. Representation of actin profiles upon ICAM binding. $CD4^+$ T cells were activated with anti-CD3/CD46 antibodies for 5 days, and were plated on precoated ICAM-1 for 10 min before fixation and staining for F-actin (green), phospho-tyrosine (pTyr, pink), and nuclei (blue). Representation of actin profiles in a 40x confocal image of a representative $CD4^+$ T cell. Each actin profile corresponds to a segment of F-actin staining from different regions of the cell, starting from the inner side of the plasma membrane (Profile 1/5), through the middle of the cytoplasm (Profile 2/5), the outer side (Profile 3/5), the inner side of the nuclear membrane (Profile 4/5), until the center of the nucleus (Profile 5/5). Scale bar: 10 µm.

Supplemental Figure 11

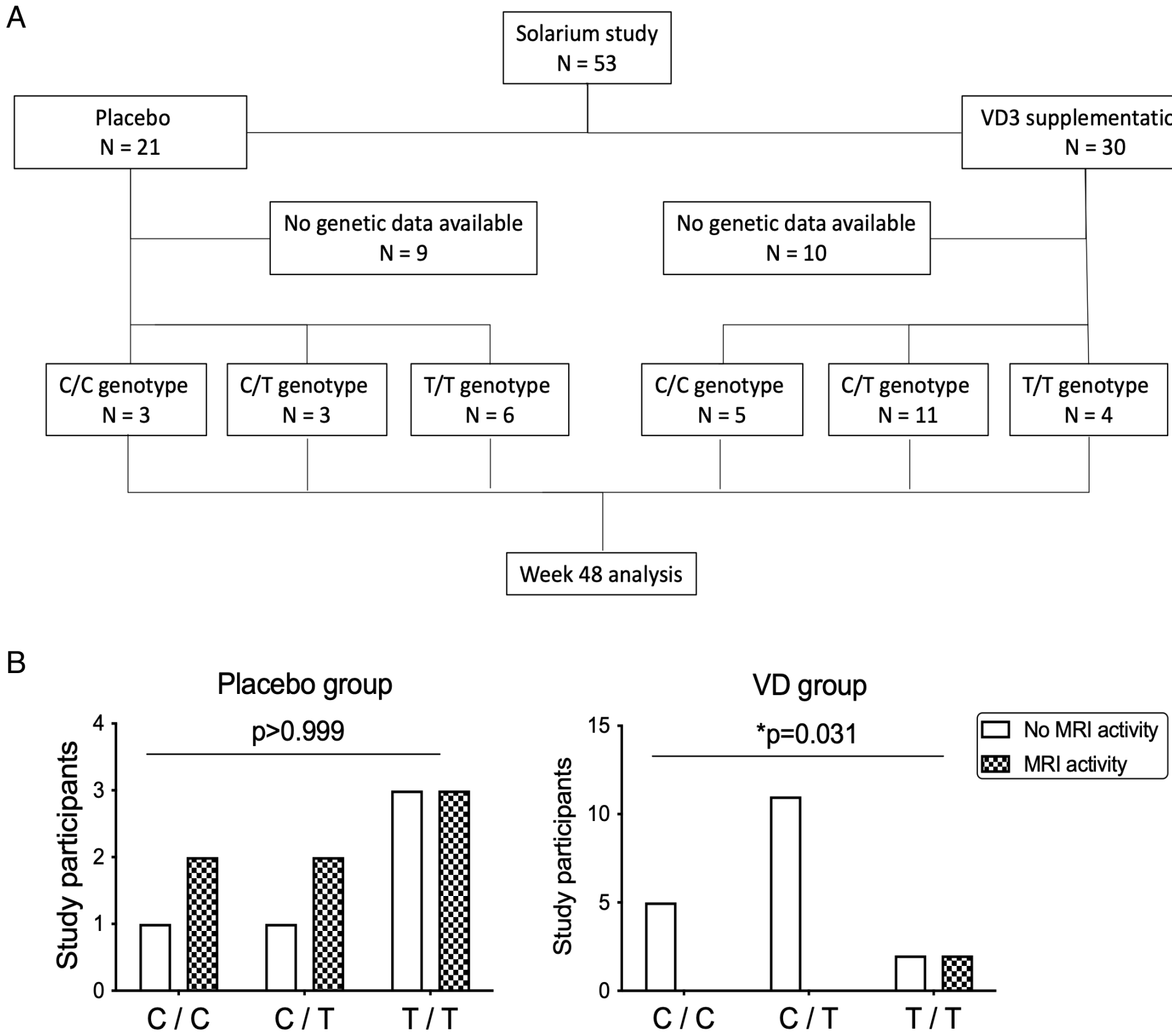

Supplemental Figure 11. Stratification of response to VD supplementation in MS by the *CD226* risk allele. People with MS treated by IFNβ and supplemented by VD or placebo for 48 weeks from the SOLARIUM trial (52) were stratified according to the *CD226* risk allele when genetic material was available. MRI activities (gadolinium-enhancing T1 and/or new or enlarging T2 lesions during 48-weeks follow-up) according to the genotype of the study participants. Data were analyzed using the Fischer test, two sided.

Supplemental Figure 12

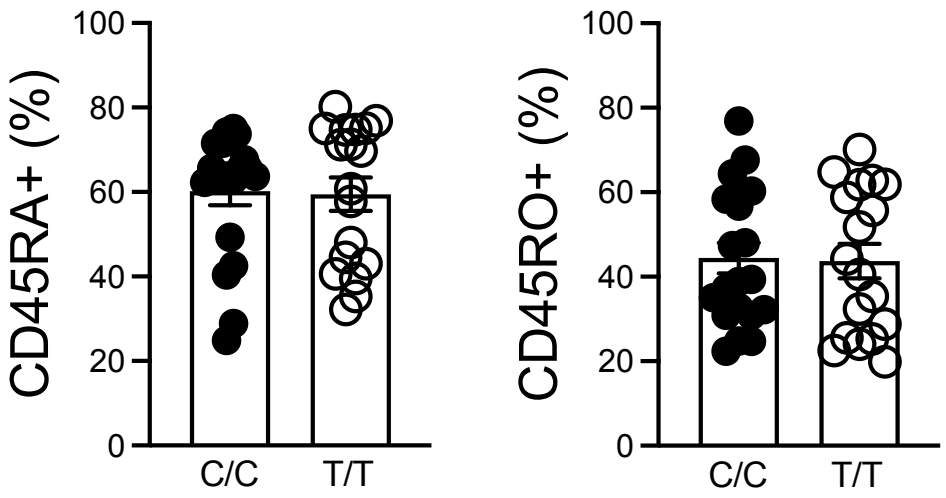

Supplemental Figure 12. Frequency of naïve and memory CD4$^+$ T cells in the healthy donors homozygous for the *CD226* C/C (n=19) or T/T (n=16) variant used in this study. Mean ± SEM, Mann Whitney test.

Supplemental Figure 13

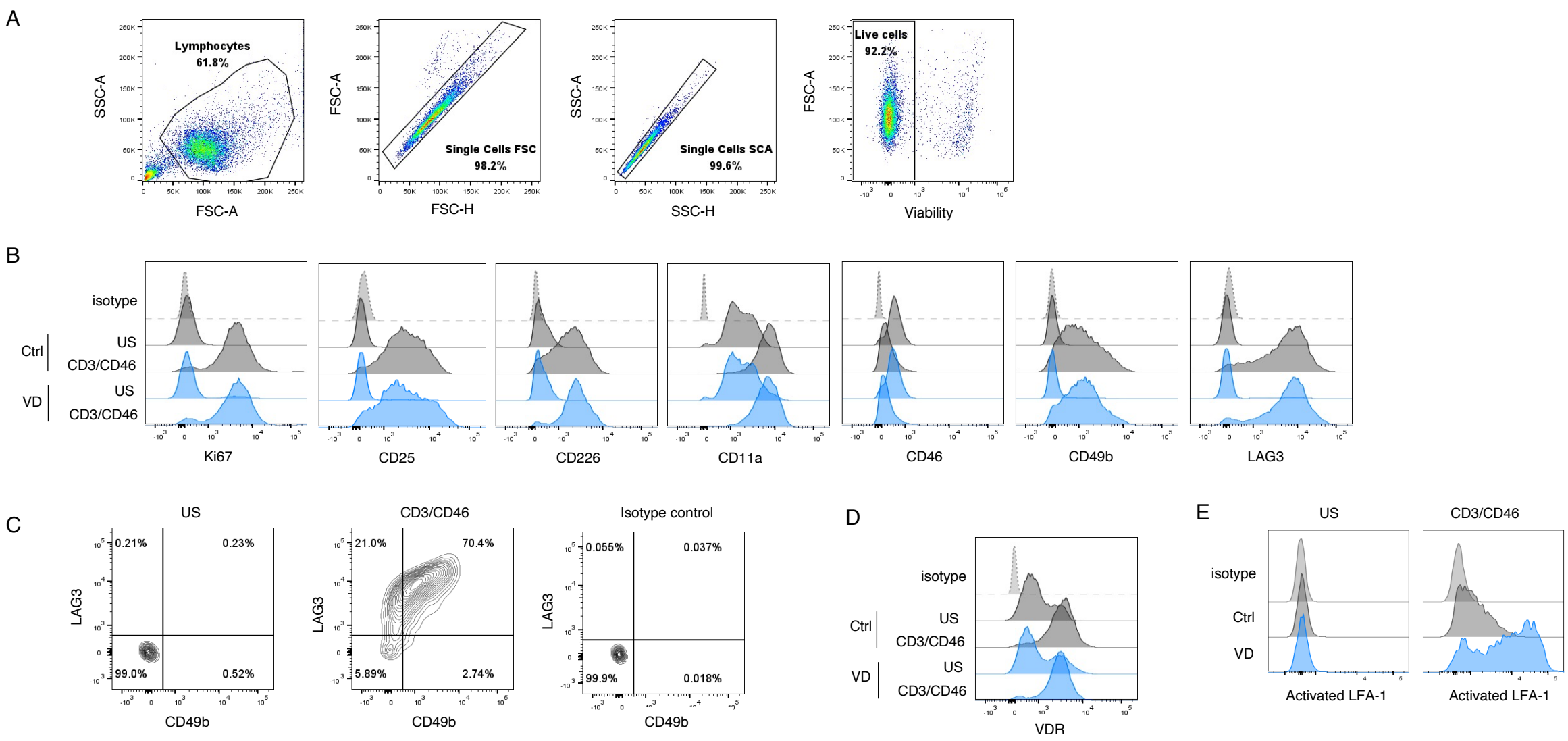

Supplemental Figure 13. Gating strategies and example of staining obtained to characterize the phenotype of the cells. (**A**) Gating strategies by gating on singlets and then on live cells. (**B**) CD4$^+$ T cells from HD homozygous for the *CD226* rs763361 C/C allele were left unstimulated (US) or activated with anti-CD3/CD46 for 5 d with (VD) or without (Ctrl) 1,25OH. Representative histograms for Ki67, CD25, CD226, CD11a, CD46, CD49b and LAG3. (**C**) Percentage of Tr1 cells based on co-expression of CD49b and LAG3. (**D**) Expression of VDR, (**E**) Expression of activated LFA-1 using the m24 Ab.

Supplemental Figure 14

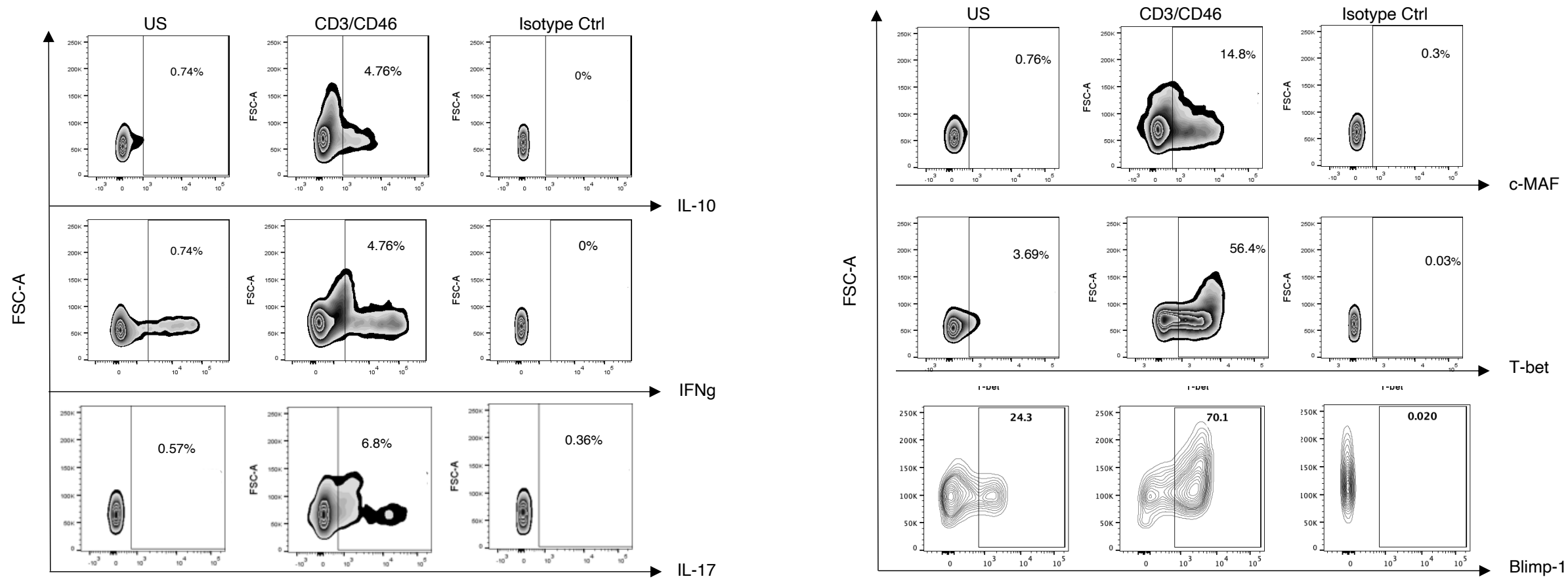

Supplemental Figure 14. Staining obtained to characterize cytokine production and expression of transcription factors. CD4+ T cells from HD homozygous for the *CD226* rs763361 C/C allele were left unstimulated (US) or activated with anti-CD3/CD46 for 3 days. Representative dot-plots for IL-10, IFNγ, IL-17 (left panels) as well as c-MAF, T-bet and Blimp-1 (right panels).

Supplemental Figure 15

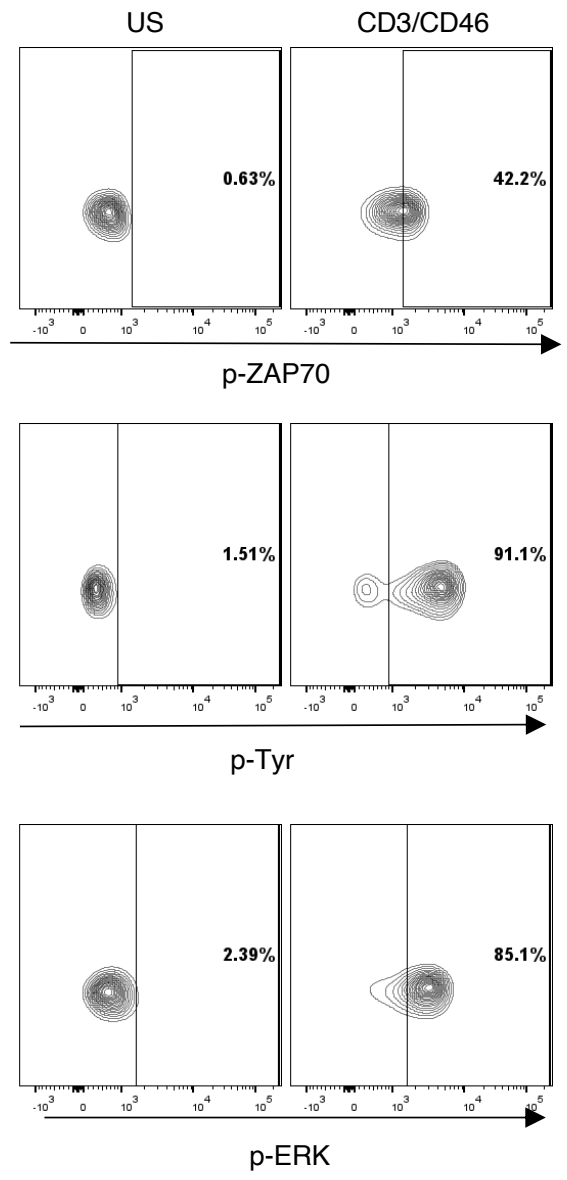

Supplemental Figure 15. Staining obtained to characterize early TCR signaling. $CD4^+$ T cells were left unstimulated or were activated for 2 min by anti-CD3/CD46 antibodies and phosphorylation of tyrosine (p-Tyr), ZAP70 (p-ZAP70) and ERK (p-ERK) assessed by flow cytometry. Representative dot-plots are shown.
